# HDGF Knockout Suppresses Colorectal Cancer Progression and Drug Resistance by Modulating the DNA Damage Response

**DOI:** 10.3390/biom15020282

**Published:** 2025-02-14

**Authors:** Riya Su, Qin Wang, Qun Hu, Wendurige, Kexin Li, Changshan Wang, Liang Tao

**Affiliations:** 1 Department of Pharmacology, Zhongshan School of Medicine, Sun Yat-sen University, Guangzhou 510080, China; 2 Department of Oncology, The Affiliated Hospital of Inner Mongolia Medical University, Hohhot 010030, China; 3 The State Key Laboratory of Reproductive Regulation and Breeding of Grassland Livestock, School of Life Sciences, Inner Mongolia University, Hohhot 010020, China

**Keywords:** HDGF, colorectal cancer, DNA damage response, homologous recombination, H3K36me3

## Abstract

Colorectal cancer (CRC) is a highly heterogeneous gastrointestinal malignancy. Despite significant advances in molecular targeted therapies for CRC in recent years, the increase in the overall survival rates for CRC patients remains limited. Therefore, there is an urgent need to explore novel drug targets. Herein, we show that heparin binding growth factor (HDGF) is highly expressed in CRC, and that its overexpression is associated with a poor disease-free interval. Additionally, we reveal that HDGF knockout reduces proliferation, migration, and invasion, while enhancing apoptosis in CRC cells, thereby validating HDGF as a potential therapeutic target for CRC. Mechanistically, we found that HDGF modulates DNA damage response and, by recruiting C-terminal binding protein-interacting protein (CtIP), it facilitates homologous recombination repair to influence CRC drug sensitivity. Furthermore, we propose that HDGF may serve as a recognition protein for H3K36me3, participating in the repair of damaged transcriptionally active genes, thus maintaining genomic stability in CRC.

## 1. Introduction

Colorectal cancer (CRC) is the most common malignant tumor type of the digestive tract, accounting for approximately 10% of the cancer diagnoses and cancer-related deaths reported annually worldwide [1]. Among common cancers, CRC ranks third in incidence and second in mortality, with these rates varying between geographical regions [2]. As developing countries progress, the global incidence of CRC is expected to rise to 2.5 million cases by 2035 [1]. Among patients diagnosed with metastatic CRC, the 1-, 3-, and 5-year survival rates are 70–75%, 30–35%, and <20%, respectively [3]. Although various treatment strategies, including surgical resection combined with chemotherapy, radiotherapy, or targeted therapy, are currently used for CRC, the overall survival (OS) rate of these patients remains poor. Therefore, an exploration of new drug targets and novel combined therapeutic approaches is urgently needed.

Cells are constantly confronted with DNA damage due to exogenous and endogenous stressors. In response to this constant threat to genomic integrity, cells have evolved sophisticated and coordinated DNA damage response (DDR) mechanisms, which include DNA damage detection, signal transduction, and damage repair. Ataxia-telangiectasia mutated (ATM) protein, ATM and Rad3-related (ATR) protein, and DNA-dependent protein kinase (DNA-PK) are members of the phosphatidylinositol 3-kinase related protein kinases (PIKK) protein family, and are the main sensors for DDR. These proteins can be recruited and activated by DNA double-strand breaks (DSBs) or replication protein A (RPA)-coated single-stranded DNA (ssDNA) [4,5]. When cells undergo DSBs, ATM, ATR, and DNA-PK initiate DDR through a series of kinase reactions, targeting numerous dual-function proteins, which serve as pivotal nodes in determining cell survival or death. These proteins orchestrate downstream checkpoints to halt cell cycle progression and recruit DNA repair proteins to the damage sites, facilitating non-homologous end-joining (NHEJ) or homologous recombination (HR) to enhance DSB repairs. Cells that are unable to undergo repair progress toward permanent arrest or programmed cell death [6].

Hepatoma-derived growth factor (HDGF), a heparin-binding protein isolated from the conditioned medium of the liver cancer-derived cell line HUH-7 [7], is present in various tissues, including the liver [8], intestines [9], retina [10], kidneys [11], and spinal cord [12]. HDGF assumes a pivotal role in embryonic development, as well as post-injury repair and regeneration within these tissues [9,12,13]. Recent research indicates that HDGF is abnormally expressed in many types of cancer [14,15,16], where it promotes tumor progression by regulating biological processes such as proliferation and differentiation [15], metastasis [17], apoptosis [18], angiogenesis [15], and therapeutic resistance [19]. Thus far, research into the mechanisms by which HDGF promotes CRC progression is limited. It has been reported that members of the HDGF family, namely, lens epithelium-derived growth factor (LEDGF), HDGF-related protein 2 (HDGFRP2), and HDGFRP3, bind histone markers through their PWWP domain. This process facilitates the recruitment of DNA damage repair factors to DSB sites, thereby promoting NHEJ or HR repair [20,21,22]. Hence, HDGF family members may act as “readers” of histone markers, recognizing these markers and recruiting damage repair factors to collectively maintain genomic stability.

However, the role of HDGF in regulating DDR and its role in CRC remain unclear. Given that HDGF family members possess a highly conserved PWWP structural domain [23], we hypothesize that HDGF has a similar function in regulating DDR. In this study, we knocked out HDGF in CRC cell lines to investigate its biological function and to further explore its effects on the DDR and DNA damage repair. This work provides novel insights into the mechanisms by which HDGF promotes CRC development. Based on the findings, we propose novel strategies for targeting HDGF in combination with chemotherapy and poly (ADP-ribose) polymerase (PARP) inhibitors for the treatment of CRC.

## 2. Materials and Methods

### 2.1. Bioinformatic Analyses

Sequencing data sets and related clinical information of patients with CRC were obtained from the Cancer Genome Atlas (TCGA). We conducted survival analysis using the R packages “survival”, “survminer”, and “ggsurvplot”.

Additionally, we obtained protein and RNA sequencing data for patients with CRC from the Clinical Proteomic Tumor Analysis Consortium (CPTAC) and the TCGA database. The “ggpubr” package was used to perform Wilcoxon tests and to generate box plots for the normal and cancer groups.

### 2.2. Clinical Tissue Samples

A total of 45 pairs of paraffin-embedded tissue samples of patients with CRC and paracancerous tissue samples were obtained from the Department of Pathology, the Affiliated Hospital of Inner Mongolia Medical University (Hohhot, China) between January and December 2017. All patients and their corresponding tissue samples had previously been confirmed by pathology. These patients had not received chemotherapy or radiotherapy during the pre-operative period. This study was approved by the local Research Ethics Committee of the Affiliated Hospital of Inner Mongolia Medical University.

### 2.3. Immunohistochemical (IHC) Staining

Tissues were cut to 3–4 μm thickness from archival formalin-fixed paraffin-embedded samples. The sections were incubated with primary antibody overnight at 4 °C. Following washing in phosphate-buffered saline, slides were incubated with goat anti-rabbit immunoglobulin G-horseradish peroxidase (IgG-HRP; GB23303; Servicebio, Wuhan, China) for 30 min at 37 °C, and the reaction was visualized with DAB reagents (ZLI-9018; ZSGB-Bio, Beijing, China) according to the instructions provided by the manufacturers. Finally, all sections were counterstained with hematoxylin. Tissue sections were examined under a light microscope, and IHC samples were further analyzed semi-quantitatively using the H-score metric. Briefly, the H-score is the sum of the percentage of stained tumor cells multiplied by an ordinal value corresponding to the intensity (0 = none, 1 = 1+, 2 = 2+, and 3 = 3+), and ranges from 0 to 300.

### 2.4. Cell Culture

Human wild-type (WT) CRC cell lines HCT116 and HT29 were obtained from The American Type Culture Collection (ATCC, Manassas, VA, USA). Both cell lines were cultured in Dulbecco’s modified Eagle’s medium (high glucose) supplemented with 10% fetal bovine serum, 100 U/mL penicillin, and 100 U/mL streptomycin at 37 °C in an atmosphere containing 5% CO_2_.

### 2.5. Generation of HDGF-Knockout (HDGF-KO) Cell Lines

For HDGF KOs, the exon 3 region of HDGF was selected to be targeted by CRISPR/Cas9 genome editing. The pair of oligos for two targeting sites was annealed and ligated to the YKO-RP003 vector (Ubigene Biosciences Co., Ltd., Guangzhou, China). The YKO-RP003-hHDGF plasmids containing each of the target single-guide RNA (sgRNA) sequences were transfected into cells by electroporation (MPK-10025; Thermo Fisher Scientific, Waltham, MA, USA). Next, 24–48 h after the transfection, puromycin was added for antibiotic selection. Subsequently, a certain number of cells were diluted using the limited dilution method and inoculated into 96-well plates. The selection of single clones was performed after 2–4 weeks, and the selected HDGF-KO clones were validated by polymerase chain reaction and Sanger sequencing. The used sgRNAs for CRISPR design were gRNA 3: TGGATTCCTCGTAAGGGAAGAGG and gRNA 4: CGA-GAACAACCCTACTGTCAAGG.

### 2.6. Colony Formation Assay

To determine the colony formation ability, 1 × 10^3^ cells were seeded into six-well plates in 2 mL of medium and cultured for 10 days until the cell colonies could be observed macroscopically. Cells were fixed with 4% paraformaldehyde for 20 min and stained with 0.1% crystal violet for 30 min.

### 2.7. Transwell Cell Migration and Invasion Assay

For the migration assays, cell suspensions were prepared at a concentration of 4–10 × 10^4^ cells in 200 μL of serum-free medium. Next, 200 μL of each cell suspension was added into polycarbonate membrane inserts (Corning Inc., Corning, NY, USA), and the chambers were placed into the pores of 24-well plates containing 600 μL of medium supplemented with 10% fetal bovine serum. For the invasion assays, the inserts were coated with Matrigel (356234; Corning Inc.) according to the instructions provided by the manufacturer; the remaining steps were the same as those for the migration assay. Following 48 h of incubation, the chambers were fixed using 4% paraformaldehyde. The cells on the upper chamber were removed, and those attached on the lower chamber were stained with crystal violet. Cells were counted in at least five random fields and three independent experiments were performed.

### 2.8. Flow Cytometry

For the apoptosis assay, cells were harvested and stained using an Annexin V-fluorescein isothiocyanate (Annexin V-FITC) Apoptosis Detection Kit (C1062; Beyotime, Shanghai, China) following the instructions provided by the manufacturer. Briefly, a total of 1 × 10^5^ cells in 195 μL of Annexin V-FITC binding buffer were transferred to a flow tube, and 5 μL of Annexin V-FITC and 10 μL of propidium iodide were added. After incubation for 15 min at room temperature in the dark, the cells were analyzed by flow cytometry.

### 2.9. Small-Interfering RNA (siRNA) Transfection

Cells were transfected with siRNA or the negative control siRNA using lipofectamine RNAIMAX (Invitrogen, Carlsbad, CA, USA) according to the instructions provided by the manufacturer. Briefly, the Lipofectamine RNAIMAX reagent and siRNAs were diluted with serum-free Dulbecco’s modified Eagle’s medium. Thereafter, the diluted reagents were mixed, incubated for 5 min at room temperature, and added to the cells. The cells were incubated for 72 h prior to western blot analysis. The sequences of the siRNAs used were as follows: HDGF Human siRNA-1 (5′-GAACGAGAAAGGAGCGUUGAAdTdT-3′) and HDGF Human siRNA-2 (5′-CGAAACAACCCUACUGUCAAdTdT-3′).

### 2.10. Immunofluorescence (IF) Assay

For γ-H2AX and RPA2 staining, cells were fixed with precooled methanol for 15 min at −20 °C. For C-terminal binding protein-interacting protein (CtIP) staining, cells were fixed with 4% paraformaldehyde for 15 min at room temperature. After fixation, the cells were blocked in blocking buffer (1× phosphate-buffered saline, 5% goat serum, 0.3% Triton X-100) for 1 h at room temperature. Subsequently, the cells were incubated with primary antibody overnight at 4 °C, followed by incubation with secondary antibodies for 1 h at room temperature. Coverslips were mounted using an antifade mountant with 4′,6-diamidino-2-phenylindole (DAPI; Thermo Fisher Scientific), and the cells were imaged using a Nikon A1R HD25 confocal microscope.

### 2.11. Immunoprecipitation (IP) and Immunoblotting (IB) Analysis

For the IP of the Ku70, Ku80, RPA2, DNA-PKcs, and MRE11-RAD50-NBS1 (MRN) complex, cells were lysed using the Pierce™ IP lysis buffer (87787; Thermo Fisher Scientific). For the IP of CtIP, nuclear extracts of cells were prepared essentially following the instructions provided for the Nuclear Complex Co-IP kit (54001; Active Motif, Carlsbad, CA, USA); of note, they were diluted in Pierce™ IP lysis buffer (Thermo Fisher Scientific). The lysis buffer was supplemented with protease and phosphatase inhibitor cocktails (78442; Thermo Fisher Scientific). Magnetic beads (88803; Thermo Fisher Scientific) were pre-incubated with anti-HDGF, anti-CtIP, or IgG control antibodies for 3 h, followed by incubation with cell lysate overnight at 4 °C. After washing four times with lysis buffer, the beads were suspended in 1× sample buffer and subjected to IB analysis. For H3K36me3 IP, we used the Immunoprecipitation Kit with Protein A+G Magnetic Beads (P2179; Beyotime) according to the instructions provided by the manufacturer.

For IB analysis, cell lysates were boiled for 10 min with 1× sample buffer. Proteins were subsequently separated by sodium dodecyl sulfate-polyacrylamide gel electrophoresis using the Mini-PROTEAN@Tetra system (BIO-RAD, Hercules, CA, USA). Next, the separated proteins were transferred onto a polyvinylidene difluoride membrane (IPFL00010; Millipore, Billerica, MA, USA) and incubated with the primary antibodies. Proteins were visualized using DyLight 800 4X PEG fluorescent dye conjugated secondary antibodies on an infrared imaging system (Odyssey CLx; LI-COR Biosciences, Lincoln, NE, USA).

### 2.12. Nude Mice Xenograft Assay

Female BALB/c nude mice (age: 6–8 weeks) were purchased from Beijing Vital River Laboratory Animal Technology Co., Ltd. (Beijing, China). All experimental procedures were approved by the Bioethics Committee of Inner Mongolia University and were conducted in compliance with ethical principles. A total of 2 × 10^6^ HT29 WT and HT29 HDGF-KO cells were subcutaneously injected into the right flanks of nude mice. On day 6 post inoculation, the mice were randomly divided into four groups: HT29 WT + saline solution; HT29 WT + treatment; HT29 HDGF-KO + saline solution; and HT29 HDGF-KO + treatment group (five mice per group). Every 3 days, the mice received intraperitoneal injections of 5-fluorouracil (5-FU) at 30 mg/kg or oxaliplatin at 10 mg/kg, and their body weights and tumor volumes were recorded. On day 21 post inoculation, the mice were euthanized, the tumors were removed and photographed, and the tumor weights were recorded.

Thereafter, the tumor tissues were fixed, dehydrated, and embedded in paraffin. Following the previously described procedures, Ki67 (PTM-5032; PTM BIO, Hangzhou, China) IHC staining was performed. The In Situ Cell Death Detection Kit (11684795910; Roche, Basel, Switzerland) was used according to the instructions provided by the manufacturer for terminal deoxynucleotidyl transferase UTP nick-end labeling (TUNEL) staining, and the slides were scanned using a digital slide scanner (Pannoramic 250; 3DHISTECH Ltd., Budapest, Hungary).

### 2.13. In Situ Proximity Ligation Assay (PLA)

HT29 cells were cultured on coverslips, fixed with precooled methanol for 15 min at −20 °C, permeabilized with 0.3% Triton X-100 for 5 min, and placed in blocking buffer for 1 h. For the visualization of protein interactions, the samples were incubated with the primary antibodies overnight at 4 °C. In situ PLAs were subsequently performed according to the instructions provided by the manufacturer (DUO92101; Sigma–Aldrich, St. Louis, MO, USA) using PLA probe anti-mouse MINUS and PLA probe anti-rabbit PLUS.

### 2.14. Chromatin-IP Sequencing (ChIP-seq)

ChIP was performed using a SimpleChIP Plus Enzymatic Chromatin IP Kit (9005S; Cell Signaling Technology, Danvers, MA, USA). Briefly, the cells were crosslinked in culture medium containing 1% formaldehyde at room temperature for 10 min, and the reaction was stopped by adding glycine solution. Subsequently, the cells were lysed, and nuclei were collected and treated with micrococcal nuclease for 20 min at 37 °C. Following the termination of the reaction with 0.05 M ethylene glycol tetraacetic acid, samples were sonicated to disrupt the nuclear membranes. Next, the samples were centrifuged and the supernatants were collected. Chromatin solutions were subsequently incubated with HDGF (11344, 5 µg/per ChIP; Proteintech, Rosemont, IL, USA) and H3K36me3 (4909, 1:50; Cell Signaling Technology) antibodies overnight at 4 °C, followed by incubation with Protein G magnetic beads for 2 h at 4 °C. The beads were washed, and chromatin was eluted. The crosslinks were reverted according to the instructions provided by the manufacturer. DNA was subsequently purified and used for ChIP-seq analysis on the Illumina Novaseq 6000 platform (Illumina Inc., San Diego, CA, USA).

The peaks of each sample were annotated by ChIPseeker [24]. The ChIP-seq signals of all samples within the gene body and its upstream and downstream 3 kb region were mapped using R software. Annotation and enrichment analysis were performed using clusterProfiler [25] and the Gene Ontology (GO) and Kyoto Encyclopedia of Genes and Genomes (KEGG) databases.

### 2.15. Statistical Analyses

Experiments were carried out at least in triplicates. Data are expressed as the mean ± standard deviation. Statistical analyses were performed with GraphPad Prism 9.3.1. (GraphPad Software Inc., San Diego, CA, USA). *p*-values < 0.05 denoted statistical significance for comparisons between experimental groups. Statistical differences among groups were analyzed by analysis of variance and Student’s *t*-test.

## 3. Results

### 3.1. HDGF KO Inhibited CRC Pathology

**Figure 1:**
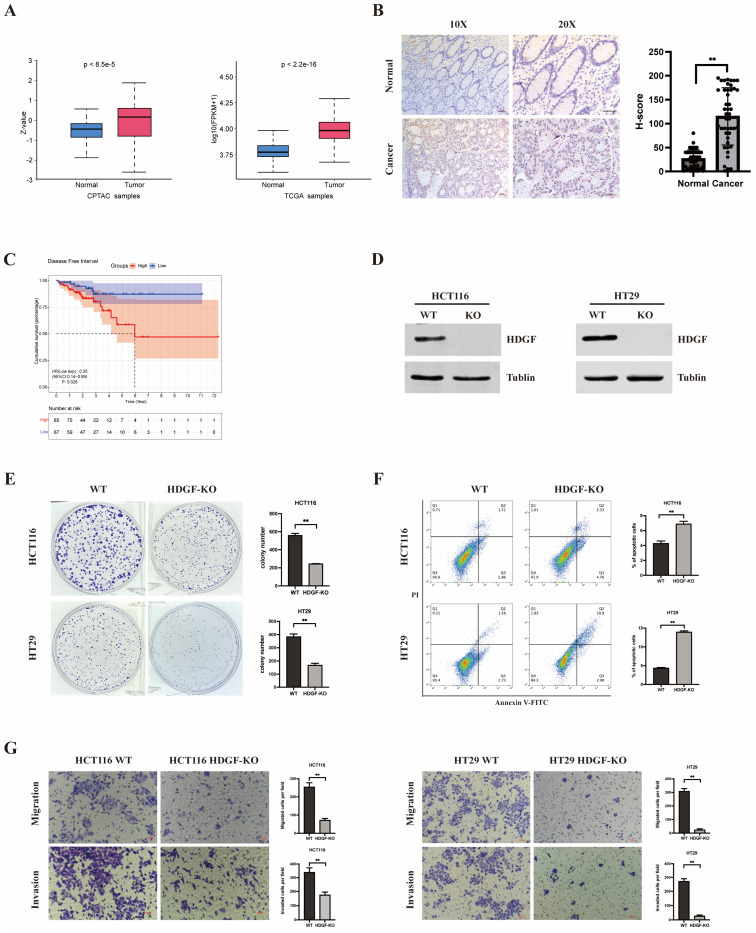
HDGF promotes the progression of CRC. (**A**) Analysis of HDGF protein and mRNA expression in adjacent normal tissues (CPTAC: n = 100, TCGA: n = 51) compared with CRC tissues (CPTAC: n = 97, TCGA: n = 615). (**B**) Evaluation of HDGF expression differences between adjacent normal tissue (n = 45) and CRC tissue (n = 45) in paraffin-embedded CRC specimens. Scale bars: 50 µm. (**C**) Analysis of association between HDGF mRNA expression levels and DFI in patients with CRC. (**D**) IB analysis confirming the efficiency of HDGF KO in HCT116 and HT29 cell lines (n = 3). (**E**–**G**) Colony formation ability, apoptosis rates, migratory and invasive abilities of HCT116 WT cells, HCT116 HDGF-KO cells, HT29 WT cells, and HT29 HDGF-KO cells (n = 3 each). Scale bars (Transwell assays): 100 µm. Data are presented as the mean ± SD. ** *p* < 0.01. Original images of (**D**) can be found in Supplementary Materials.

To assess the clinical relevance of HDGF in patients with CRC, proteomic and transcriptomic data from the CPTAC and TCGA databases were analyzed to compare HDGF expression in CRC tissues and adjacent normal tissues (Figure 1A). The results indicate a significant increase in HDGF protein and mRNA expression levels in CRC tissues. IHC staining on paraffin-embedded CRC specimens further confirmed the significantly higher HDGF protein levels in CRC tissues (Figure 1B). Additionally, survival analysis using the TCGA transcriptome data set highlighted a negative correlation between high HDGF expression and disease-free interval, suggesting a strong association between elevated HDGF expression and CRC progression (Figure 1C).

To elucidate the biological role of HDGF in CRC, CRISPR/Cas9-based gene editing was used to target the third exon of HDGF for KO (Figure S1A). Polymerase chain reaction amplification and the sequencing of genomic DNA from the modified cells confirmed the successful KO of 92 base pairs in this region compared with WT DNA (Figure S1B). The IB results show that the HDGF protein expression levels were significantly lower in HCT116 HDGF-KO and HT29 HDGF-KO cells versus HCT116 WT and HT29 WT cells, indicating the successful KO of HDGF (Figure 1D). Subsequently, we performed colony formation assays, Transwell assays, and flow cytometry in HCT116 WT and HCT116 HDGF-KO cells, as well as HT29 WT and HT29 HDGF-KO cells (Figure 1E–G). We found that HDGF KO inhibited the proliferation, migration, and invasion of CRC cells, while promoting apoptosis. Additionally, we assessed the apoptosis rate induced by non-specific sgRNA; this rate was similar to that observed in WT cells (Figure S1C). This finding confirms that the observed promotion of apoptosis is due to the HDGF KO itself, rather than the CRISPR/Cas9 system.

### 3.2. HDGF KO Activated DDR and Induced DNA-PK and ATM-Mediated Phosphorylation of p53

**Figure 2:**
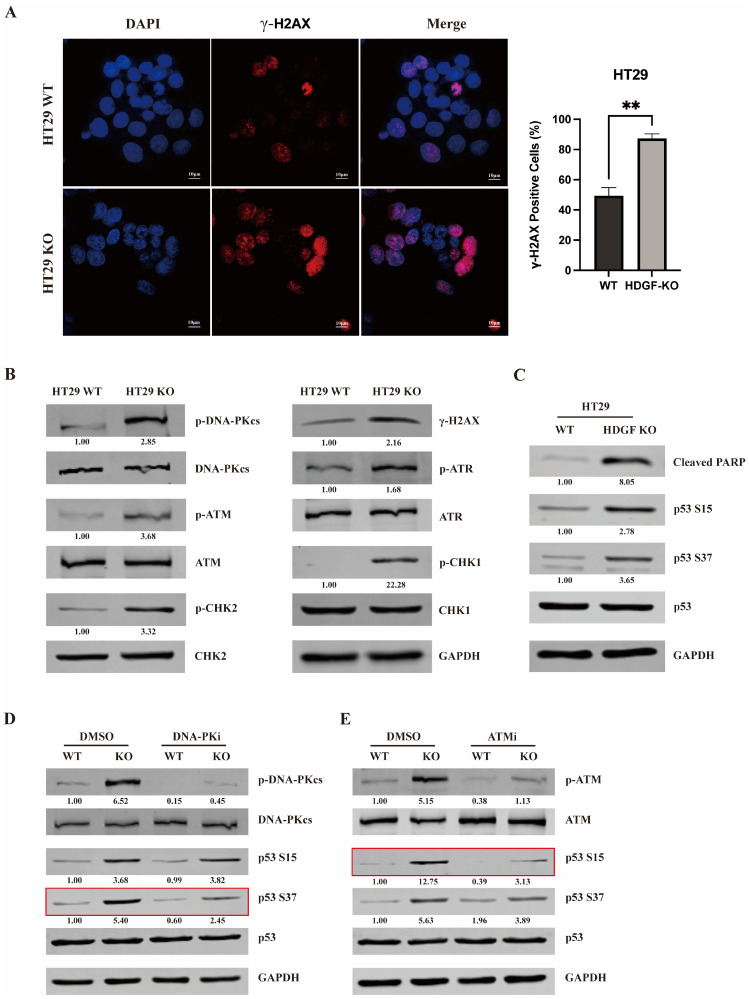
HDGF KO activates DDR and induces DNA-PK and the ATM-mediated phosphorylation of p53. (**A**) IF analysis of γ-H2AX expression in HT29 WT and HT29 HDGF-KO cells (n = 3). Scale bars: 20 µm. (**B**) IB assessment of phosphorylated DNA-PKcs, ATM, ATR, CHK1, and CHK2 in HT29 WT and HT29 HDGF-KO cells (n = 3). (**C**) IB detection of Cleaved-PARP, p53 Ser15 and Ser37 phosphorylation in HT29 WT and HT29 HDGF-KO cells (n = 3). (**D**,**E**) IB detection of changes in p53 Ser37 and Ser15 phosphorylation after treatment with 10 µM Nu7441 or 10 µM KU-55933 for 24 h in HT29 WT and HT29 HDGF-KO cells (n = 3). The data are presented as the mean ± SD. ** *p* < 0.01. Original images of (**B**–**D**) can be found in Supplementary Materials.

The phosphorylated form of histone H2AX at serine 139, γ-H2AX, serves as a crucial marker of DSBs [26]. To assess the involvement of HDGF in DDR, γ-H2AX expression in HT29 WT and HT29 HDGF-KO cells was examined by IF and IB. The results show a significant increase in γ-H2AX-positive cells as well as γ-H2AX protein expression in HT29 HDGF-KO cells compared with HT29 WT cells, suggesting that HDGF KO leads to the accumulation of DNA DSBs in CRC cells (Figure 2A,B). To rule out the possibility that the increased DNA DSBs were caused by the CRISPR/Cas9 system, we used siRNAs targeting HDGF to knockdown HDGF in HT29 cells. We found that HDGF knockdown also significantly increased the expression of γ-H2AX (Figure S1D). These results indicate that the DNA DSBs are caused by the HDGF KO itself, rather than by the CRISPR/Cas9. Additionally, HDGF KO markedly elevated the phosphorylation levels of DNA-PKcs (S2056 site), ATM (S1981 site), ATR (T1989 site), and their downstream targets, checkpoint kinase 1 (CHK1; S345 site) and CHK2 (Thr68 site), in HT29 cells (Figure 2B). These phosphorylation events are commonly recognized as activation markers of DDR [27], thereby confirming that HDGF KO can activate DDR.

The phosphorylation of p53 at Ser15 and Ser37 sites mediated by DNA-PK and ATM can promote p53-mediated apoptosis [28,29,30,31]. Therefore, we examined the levels of apoptotic marker, cleaved-PARP, and the phosphorylation of p53 Ser15 and Ser37 in HT29 WT and HT29 HDGF-KO cells (Figure 2C). The results show that the levels of Cleaved-PARP and the phosphorylation of p53 at Ser15 and Ser37 sites were significantly upregulated in HT29 HDGF-KO cells compared with HT29 WT cells. Subsequently, the treatment of HT29 WT and HT29 HDGF-KO cells with DNA-PK inhibitor Nu7441 and ATM inhibitor KU-55933 reversed the observed increases in p53 phosphorylation at Ser37 and Ser15, respectively, induced by HDGF KO (Figure 2D,E, highlighted with a red box). These results support the hypothesis that HDGF KO promotes p53-mediated apoptosis by inducing DNA-PK and the ATM-mediated phosphorylation of p53 in CRC.

### 3.3. HDGF Was Implicated in HR-Mediated Damage Repair

**Figure 3:**
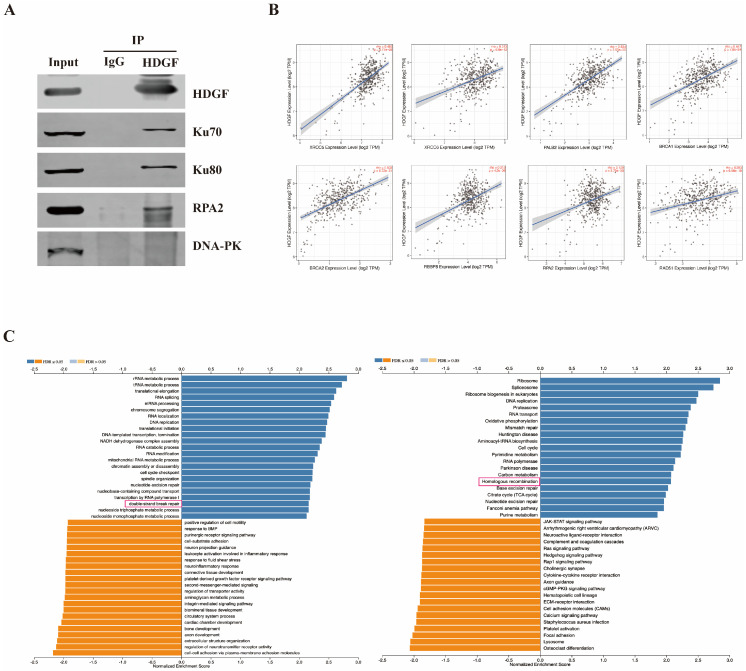
HDGF enhances HR-mediated damage repair. (**A**) Co-IP was performed to verify the endogenous interactions between HDGF and Ku70, Ku80, RPA2, and DNA-PKcs in HT29 cells (n = 3). (**B**) Correlation of HDGF expression with genes including XRCC5, XRCC6, PALB2, BRCA1, BRCA2, RBBP8, RPA2, and RAD51 in patients with CRC. The data were analyzed using the functional module of TIMER (timer.cistrome.org). (**C**) GO and KEGG enrichment analyses of the gene sets co-expressed with HDGF in patients with CRC. Data were analyzed using the functional module of LinkedOmics (http://www.linkedomics.org/login.php). Original images of (**A**) can be found in Supplementary Materials.

The Ku70-Ku80 heterodimer, a 150 kDa complex, initially identifies and binds to DNA damage sites, triggering DNA-PK-mediated NHEJ or CtIP-MRN complex-mediated HR repair [32,33]. To investigate the role of HDGF in DNA damage repair, endogenous interactions between HDGF and Ku70, Ku80, and key NHEJ and HR effectors DNA-PKcs and RPA2 were investigated in HT29 WT cells. Our findings reveal that HDGF binds Ku70, Ku80, and RPA2, but not DNA-PKcs (Figure 3A). In addition, we utilized the functional module of LinkedOmics to perform GO and KEGG enrichment analyses on the gene sets co-expressed with HDGF using TCGA transcriptome data from 379 patients with CRC (Figure 3C). We found that HDGF is positively correlated with DSB repair and the HR pathway. Further analysis using the functional module of TIMER on TCGA transcriptome data from 458 patients with CRC showed a positive correlation between HDGF levels and several HR-related genes, including X-ray repair cross complementing 5 (XRCC5; Ku80), XRCC6 (Ku70), partner and localizer of BRCA2 (PALB2), BRCA1, BRCA2, retinoblastoma-binding protein 8 (RBBP8; CtIP), RPA2, and RAD51. This evidence indicates that HDGF may promote HR-mediated damage repair (Figure 3B).

### 3.4. HDGF KO Inhibited HR

**Figure 4:**
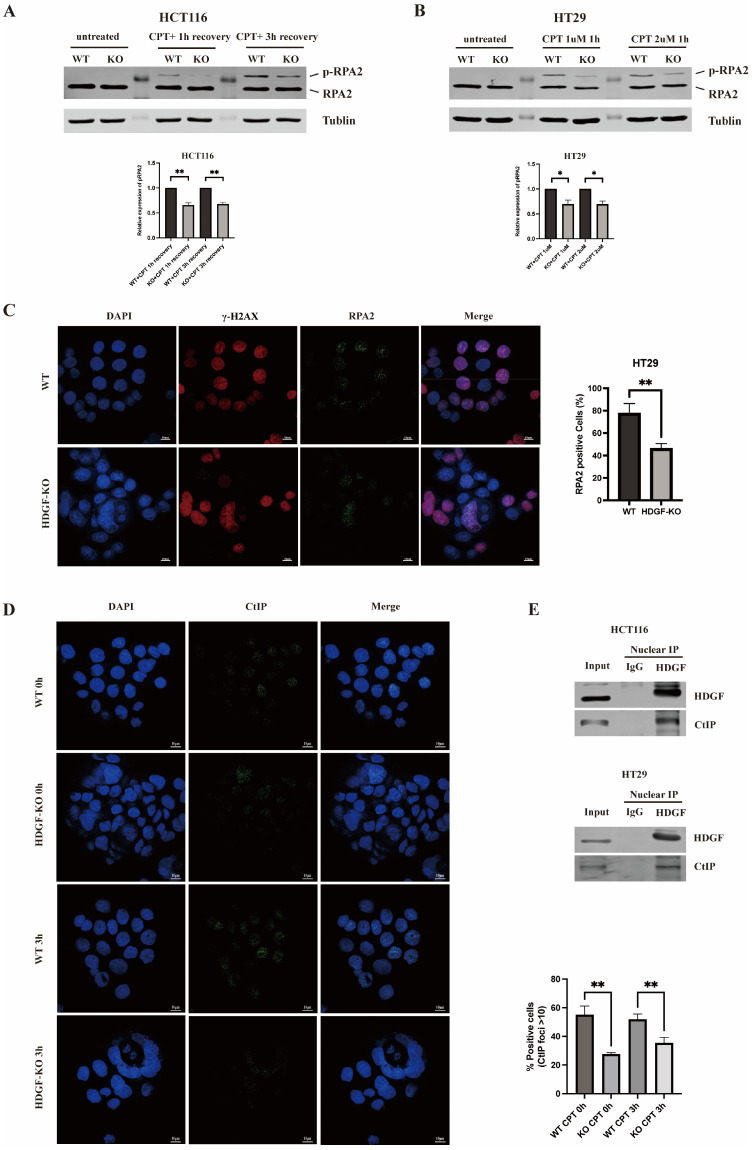
HDGF KO reduced RPA2 and CtIP accumulation at DNA DSB sites. (**A**) IB analysis of phosphorylated RPA2 levels in HCT116 WT and HCT116 HDGF-KO cells treated with 1 µM CPT for 1 h, followed by recovery for 1 h and 3 h (n = 3). (**B**) IB analysis of phosphorylated RPA2 levels in HT29 WT and HT29 HDGF-KO cells treated with 1 µM and 2 µM CPT for 1 h (n = 3). (**C**) IF detection of the proportion of γ-H2AX-positive cells with RPA2 foci in HT29 WT and HT29 HDGF-KO cells treated with 2 µM CPT for 1 h. Scale bars, 10 µm (n = 3). (**D**) IF detection of the proportion of cells with CtIP foci in HT29 WT and HT29 HDGF-KO cells treated with 2 µM CPT for 1 h, followed by recovery for 0 h and 3 h. Scale bars: 10 µm (n = 3). (**E**) Nuclear complex co-IP was performed to verify the interaction between HDGF and CtIP in the nuclei of HCT116 WT and HT29 WT cells (n = 3). Data are presented as the mean ± SD. * *p* < 0.05, ** *p* < 0.01. Original images of (**A**,**B**,**E**) can be found in Supplementary Materials.

The phosphorylation of RPA2 is a marker for the initiation of the HR pathway [34]. To clarify whether HDGF promotes HR-mediated DNA repair in CRC cells, we induced DNA DSBs in HCT116 WT and HCT116 HDGF-KO cells, as well as HT29 WT and HT29 HDGF-KO cells, using the DNA topoisomerase I inhibitor camptothecin (CPT). Subsequently, RPA2 phosphorylation levels were measured (Figure 4A,B). The results showed that HDGF KO can significantly reduce the levels of CPT-induced RPA2 phosphorylation. Additionally, we used CPT to induce DNA DSBs in HT29 WT and HT29 HDGF-KO cells, and detected RPA2 foci formation by IF. Moreover, the proportion of γ-H2AX-positive cells with RPA2 foci was significantly reduced in HT29 HDGF-KO cells compared with HT29 WT cells (*p* < 0.01) (Figure 4C). These results suggest that HDGF KO can inhibit HR activity.

### 3.5. HDGF KO Inhibited CtIP-Mediated DNA End Resection

HR is a complex repair process that is typically initiated by CtIP and MRN (MRE11-RAD50-NBS1) complex-mediated DNA end resection [35]. To determine whether HDGF promotes HR through enhancing the DNA end resection by the recruitment of CtIP, we performed nuclear co-IP assays to detect the nuclear interaction between HDGF and CtIP in HCT116 WT and HT29 WT cells (Figure 4E). The data show that HDGF interacts with CtIP in the nuclei of HCT116 WT and HT29 WT cells. Next, we used CPT to induce DNA DSBs in HT29 WT and HT29 HDGF-KO cells, and performed IF assays after recovery for 0 h and 3 h (Figure 4D). The results show that the proportion of cells with CtIP foci was significantly reduced in HT29 HDGF-KO cells compared with HT29 WT cells. These data suggest that HDGF KO inhibits HR through reduced DNA end resection by blocking CtIP recruitment.

### 3.6. HDGF KO Increased Sensitivity to Chemotherapy and PARP Inhibitors in CRC

**Figure 5:**
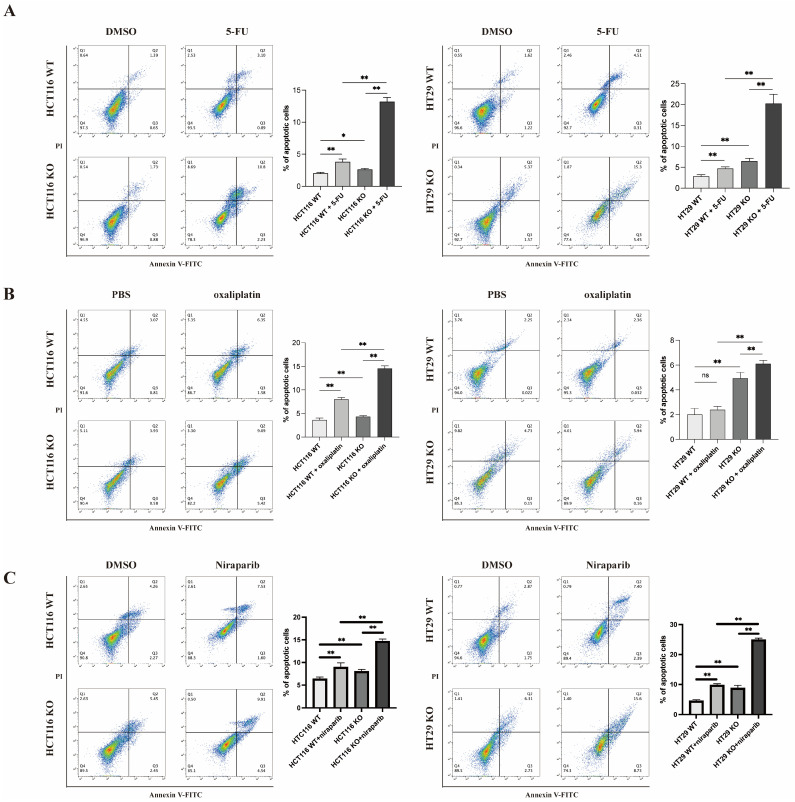
HDGF KO enhanced 5-FU-, oxaliplatin-, and niraparib-induced apoptotic effects in CRC cells. Following the treatment of HCT116 WT, HCT116 HDGF-KO, HT29 WT, and HT29 HDGF-KO cells with (**A**) 10 µM 5-FU, (**B**) 10 µM oxaliplatin, and (**C**) 10 µM niraparib for 48 h, flow cytometry analyses were performed to assess apoptosis (n = 3 each). Data are presented as the mean ± SD. * *p* < 0.05, ** *p* < 0.01. ns: no significant difference.

**Figure 6:**
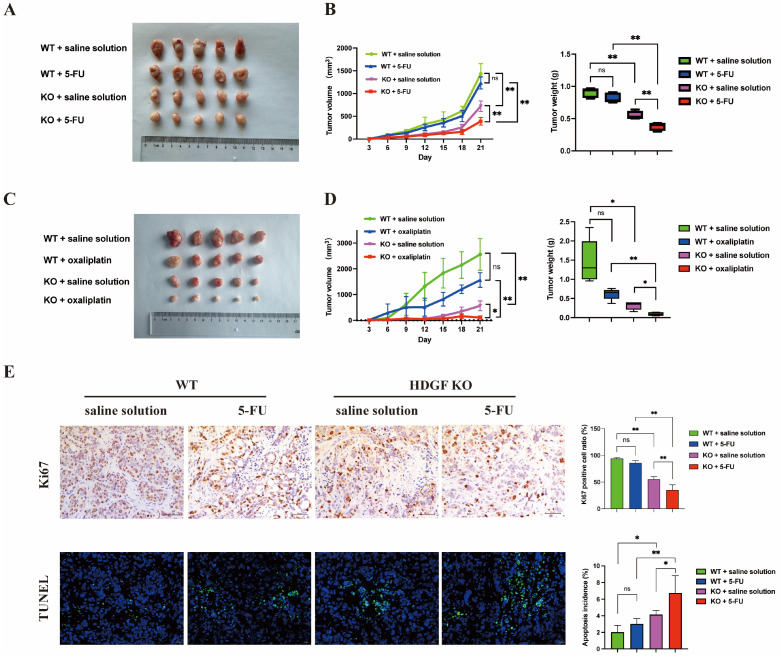
HDGF deficiency led to chemotherapy sensitivity in CRC. (**A**–**D**) Changes in tumor volume and weight from HT29 WT and HT29 HDGF-KO groups with and without 5-FU treatment (n = 5) as well as with and without oxaliplatin treatment (n = 5), respectively. (**E**) IHC and fluorescence TUNEL assays were used to assess Ki67 staining and cell apoptosis in tumor tissues of each group (n = 5). Scale bars: 50 µm (IHC) and 20 µm (TUNEL). Data are presented as the mean ± SD. * *p* < 0.05, ** *p* < 0.01. ns: no significant difference.

HR-mediated damage repair is a key mechanism for the emergence of chemotherapy resistance. To determine whether HDGF affects the sensitivity of CRC cells to chemotherapeutic agents, we treated HCT116 WT, HCT116 HDGF-KO, HT29 WT, and HT29 HDGF-KO cells with the commonly used CRC chemotherapy drugs 5-FU and oxaliplatin, and measured cell apoptosis using flow cytometry (Figure 5A,B). The results indicate that HDGF KO significantly enhances the apoptotic response to both drugs in HCT116 and HT29 cells. Furthermore, in vivo experiments showed that, compared with HT29 WT tumors, HDGF KO significantly reduced tumor volume and weight, and enhanced sensitivity to 5-FU or oxaliplatin (Figure 6A–D). Subsequently, Ki67 IHC staining and fluorescent TUNEL assay were used to analyze the proliferation and apoptosis in excised tumors (Figure 6E). The results reveal that the HDGF KO 5-FU treatment group had a significantly reduced tumor cell proliferation rate and increased tumor cell apoptosis rate compared with the WT 5-FU treatment group. These results confirm that HDGF KO can increase the sensitivity of CRC to chemotherapeutic agents.

Additionally, the activity of the HR pathways is closely related to PARP inhibitor sensitivity. Therefore, we treated all four groups of cells with niraparib. We found that HDGF KO significantly intensifies the pro-apoptosis effects of niraparib in HCT116 and HT29 cells (Figure 5C), clearly demonstrating that HDGF KO increases CRC cell sensitivity to PARP inhibitors.

### 3.7. HDGF Maintained CRC Genome Stability Through Recognition of H3K36me3

**Figure 7:**
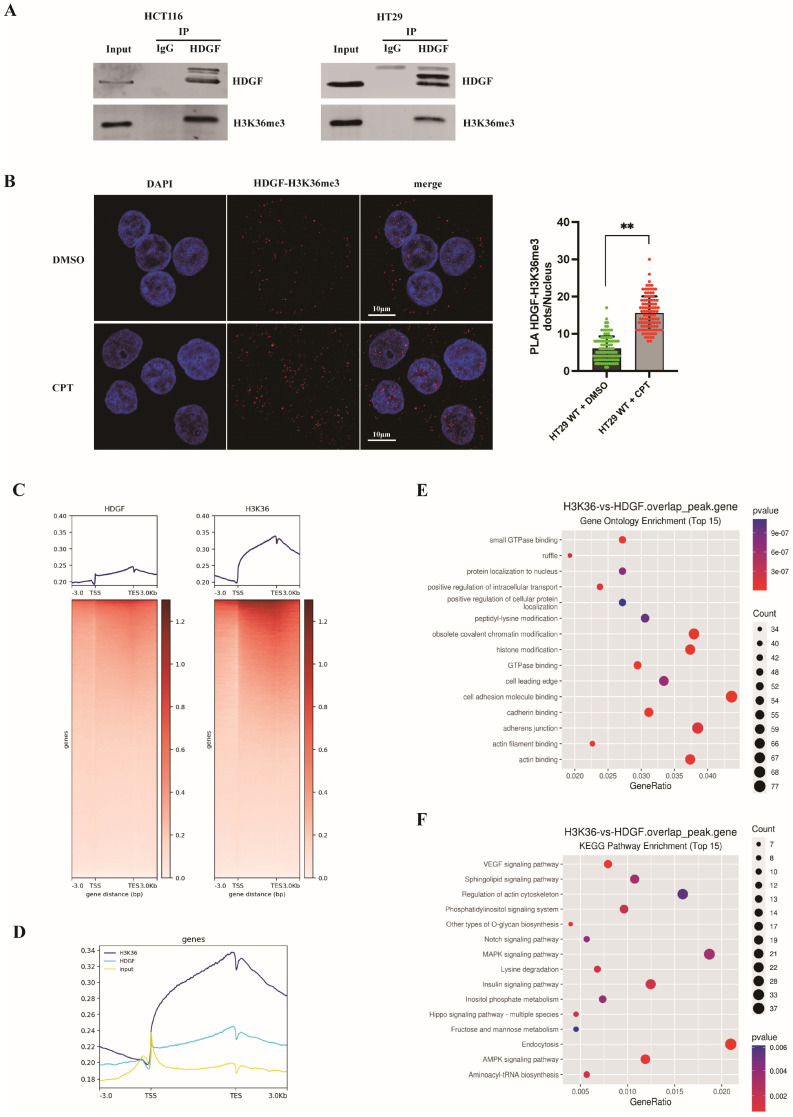
Interaction between HDGF and H3K36me3 and enrichment characteristics of their related gene sets. (**A**) Co-IP was performed to verify the interaction between HDGF and H3K36me3 in HCT116 WT and HT29 WT cells (n = 3). (**B**) PLA assays were used to study the interaction between HDGF and H3K36me3 in HT29 WT cells following treatment with and without 2 µM CPT for 1 h. Median values of nuclear PLA signals from 100 cells per condition are shown as a scatter plot with bars. Scale bars: 10 µm. (**C**) Heatmaps illustrating the distribution of HDGF and H3K36me3 on the gene body in HCT116 WT cells. (**D**) Peak map showing the distributions of HDGF and H3K36me3 on gene bodies and their upstream and downstream adjacent 3 kb regions in HCT116 WT cells. (**E**,**F**) GO and KEGG enrichment analyses of gene sets associated with overlapping peaks between HDGF and H3K36me3 ChIP-seq in HCT116 WT cells. Data are presented as the mean ± SD. ** *p* < 0.01. Original images of (**A**) can be found in Supplementary Materials.

H3K36me3, a transcriptionally active chromatin mark, plays a vital role in regulating DNA damage repair and maintaining genomic stability [36]. Studies have indicated that H3K36me3 enhances HR repair in actively transcribed genes by recruiting CtIP [20]. To investigate the relationship between HDGF and H3K36me3, co-IP experiments were conducted in HCT116 WT and HT29 WT cells (Figure 7A). The results confirm that HDGF is capable of binding to H3K36me3 in CRC cell lines. The dynamics of this interaction were further examined using the PLA assay (Figure 7B). Nuclear proximity ligation signals confirmed a significant increase in proximity between HDGF and H3K36me3 upon CPT treatment, indicating that the localization of these proteins is regulated by DNA damage.

Previous studies have shown that the distribution of H3K36me3 on gene bodies can determine the recruitment of HR repair-related factors [37]. To delineate the genomic distribution of HDGF and H3K36me3 in CRC cells, ChIP-seq experiments were performed on HCT116 cells, and the sequencing reads were used to generate heatmaps (Figure S2A and Figure 7C). The results show that HDGF and H3K36me3 signals were predominantly distributed in gene bodies. To compare and visualize this distribution, the ChIP-seq signals of HDGF and H3K36me3 were mapped near the transcription start sites (TSSs) and gene body, and their adjacent upstream and downstream 3 kb regions (Figure S2B and Figure 7D). Moreover, we confirmed the consistent trend in the distribution of HDGF and H3K36me3 across gene bodies.

To explore the genes and related functions involved in HDGF-H3K36me3-mediated DNA repair in CRC, Venn diagrams of ChIP-seq peaks for HDGF and H3K36me3 were created to first identify overlapping peaks (Figure S2C). Subsequent GO and KEGG enrichment analyses were performed on the gene sets associated with these overlapping peaks (Figure 7E,F). The constructed GO bubble plot showed that the genes corresponding to HDGF-H3K36me3 overlapping peaks were enriched in obsolete covalent chromatin modification, histone modification, cell adhesion molecule binding, adherens junction, and actin binding, among other processes. The KEGG bubble plot showed that the overlapping peaks were primarily enriched in the regulation of actin cytoskeleton and signaling pathways for mitogen-activated protein kinase (MAPK), insulin, AMP-activated protein kinase (AMPK), and vascular endothelial growth factor (VEGF), as well as other pathways. All these pathways are associated with cancer-promoting processes such as proliferation, metastasis, anti-apoptosis, drug resistance, and angiogenesis. These findings suggest that HDGF recognizes H3K36me3, leading to the repair of genes related to these pathways. Through this effect, the pathways sustain their normal activity and subsequently promote proliferation, metastasis, anti-apoptosis, drug resistance, and angiogenesis in CRC cells.

## 4. Discussion

Recent research has shown that HDGF is implicated in oncogenesis. However, the mechanisms through which HDGF promotes CRC progression remain unclear. In this study, we found that HDGF expression is significantly higher in CRC tissues than in adjacent normal tissues, and that high HDGF expression is negatively correlated with disease-free interval in patients with CRC (Figure 1A–C). Additionally, we also observed that HDGF KO in HCT116 and HT29 cells inhibited proliferation, migration, and invasion, and promoted apoptosis (Figure 1E–G). These findings indicate that HDGF promotes CRC progression and is a potential molecular target for therapeutic intervention.

The DDR is primarily controlled by three related kinases, namely, DNA-PK, ATM, and ATR. The autophosphorylation of the S2056 and T2609 sites in DNA-PKcs, the S1981 site in ATM, and the T1989 site in ATR promotes their functional activation, rendering these sites markers for the activation of each respective protein in humans [27]. In this study, we found that knocking out HDGF in CRC cells leads to the accumulation of DNA DSBs and significantly increases the phosphorylation levels of DNA-PKcs S2056, ATM S1981, ATR T1989, and their downstream targets CHK1 S345 and CHK2 Thr68 (Figure 2A,B). These results indicate that HDGF is implicated in maintaining genomic stability in CRC by regulating the DDR.

Importantly, p53 plays a crucial role in the cell fate decision of survival or death after DNA damage, with the degree of damage dictating the extent of p53 activation. This may be controlled by the p53–MDM2 feedback loop [6]. MDM2 is a negative regulator of p53. As p53 transcriptionally activates MDM2, the latter targets p53 for degradation. This alternating dominance of positive and negative feedback may cause periodic oscillations in p53 and MDM2 levels, generating pulses [31]. Minimal DNA damage results in few p53 pulses, leading to cell cycle arrest and potentially allowing time for DNA repair and cell survival. Conversely, severe DNA damage triggers continuous p53 pulses, inducing apoptosis [31,38]. In this model, phosphorylation at specific sites on p53, such as Ser15 and Ser37, mediated by ATM and DNA-PK, is crucial for inhibiting the interaction of p53 with MDM2, thereby enhancing p53-mediated apoptosis [28,29,30,31]. We found that in HT29 cells, the KO of HDGF elevated the levels of the apoptosis marker cleaved-PARP and significantly increased the phosphorylation levels of p53 Ser15 and Ser37 sites (Figure 2C). However, DNA-PK and ATM inhibitors reversed these increases in phosphorylation (Figure 2D,E). These data suggest that HDGF KO enhances p53 activity by activating DDR, thereby promoting the dissociation of the p53-MDM2 complex and subsequent apoptosis in CRC cells. Interestingly, Sasaki et al. demonstrated that endogenous p53 can transcriptionally repress HDGF expression following DNA damage in cancer cells, and this p53-mediated repression of HDGF effectively alleviated cancer progression [39]. Furthermore, they observed a significant correlation between HDGF expression and p53 mutational status, with HDGF being more frequently overexpressed in primary tumor tissues harboring mutant p53 [39]. Therefore, based on the findings reported by Sasaki et al. and our results, p53 suppresses tumor progression by downregulating HDGF, while the low expression of HDGF further enhances p53 activity. These findings further underscore the importance of HDGF as a potential therapeutic target in the treatment of cancer.

HR and NHEJ are primary repair pathways of DNA DSBs in mammalian cells. Following DNA DSBs, Ku70-Ku80 initially binds to the broken DNA ends. When cellular environments are unfavorable for DNA end resection, Ku70-Ku80 are retained at the DNA end, where they recruit DNA-PKcs to promote NHEJ [32]. Conversely, when cellular environments favor DNA end resection, the nuclease activity of MRE11 converts blunt ends into 3′ ssDNA, displacing Ku70-Ku80 from the DNA end and enabling HR repair [32]. Bremer et al. [40] and Zhao et al. [41] have found that HDGF can interact with Ku70, Ku80, and DNA-PKcs, suggesting its potential cooperation with these proteins in NHEJ repair. Consistently, we observed the endogenous binding of HDGF with Ku70 and Ku80 in CRC cells, but not with DNA-PKcs (Figure 3A). Intriguingly, our findings also revealed that HDGF can bind to the HR-related protein RPA2 (Figure 3A). Additionally, we found that HDGF is positively associated with DNA DSB repair and the HR pathway in CRC (Figure 3C), and that HDGF expression is positively correlated with the expressions of various HR-related genes, including XRCC5 (Ku80), XRCC6 (Ku70), RPA2, RAD51, RBBP8 (CtIP), BRCA1, BRCA2, and PALB2, among others (Figure 3B). These results confirm that HDGF promotes HR-mediated damage repair in CRC.

RPA2 phosphorylation recruits PALB2 and BRCA2 to RPA2-bound DNA damage sites, facilitating RAD51 assembly; thus, it serves as a marker for HR initiation [21,34,42,43]. Under conditions of CPT-induced DSBs in CRC cells, the KO of HDGF reduced RPA2 phosphorylation and the proportion of cells with RPA2 foci (Figure 4A–C), providing further evidence that HDGF supports HR in CRC. Additionally, given that HR is typically initiated by CtIP and MRN complex-mediated DNA end resection [35], we found that HDGF interacts with CtIP within the nucleus of CRC cells (Figure 4E), and that HDGF KO reduced the proportion of cells with CtIP foci (Figure 4D). These results suggest that HDGF promotes HR by facilitating DNA end resection through CtIP recruitment.

Chemotherapy remains a foundational treatment for CRC; nonetheless, resistance often develops during treatment, leading to recurrence and poor prognosis for patients with CRC. In recent years, several studies have demonstrated that HR-mediated damage repair contributes to 5-FU and oxaliplatin resistance in CRC cells [44,45,46]. Moreover, HR activity is also closely linked to the sensitivity of cells to PARP inhibitors. Arena et al. [47] observed that most CRC cell lines harboring KRAS or BRAF mutations, as well as other anti-epidermal growth factor receptor (anti-EGFR) resistance mutations, were resistant to PARP inhibitors. Among them, a few CRC cell lines sensitive to olaparib exhibit HR functional defects [47]. Furthermore, the response of CRC cell lines and patient-derived organoids to PARP blockade is positively correlated with sensitivity to oxaliplatin [47]. In fact, our results show that knocking out HDGF in CRC cells can significantly increase the pro-apoptotic effects of 5-FU, oxaliplatin, and niraparib (Figure 5A–C). In vivo experiments confirmed that, compared with using 5-FU or oxaliplatin alone, the combination of HDGF KO with 5-FU or oxaliplatin can further inhibit tumor growth in mice (Figure 6A–D). These findings suggest that targeting HDGF is a promising therapeutic strategy to overcome resistance to chemotherapy and PARP inhibitors in CRC.

H3K36me3 is a transcriptionally active histone marker that is mainly distributed in the gene bodies of transcribed active genes [48]. It plays an important role in HR-mediated DNA damage repair and the maintenance of genome stability after DNA damage [36]. In this process, proteins containing a PWWP domain act as H3K36me3 recognition proteins, primarily responsible for transmitting DNA damage signals and recruiting repair proteins to specific sites [49]. Daugaard et al. [20] discovered that LEDGF binds to H3K36me3 through its PWWP domain and concurrently binds CtIP in a DNA damage-dependent manner, thereby enhancing the binding of CtIP to active chromatin and promoting its entry into DNA DSB sites. However, HDGFRP2 tends to bind to the transcriptionally silent histone marker H3K9me3, suggesting that HDGFRP2 recruits HR repair-related factors to damaged silent genes or active genes that are silenced upon DNA damage [21]. Similarly, our findings indicate that HDGF recruits CtIP to damage sites and interacts with H3K36me3 (Figure 7A,B). Additionally, HDGF is mainly distributed on the gene bodies, which is consistent with the distribution of H3K36me3 (Figure 7C,D). This implies that HDGF recognizes H3K36me3 and subsequently recruits CtIP for damage repair in transcriptionally active genes. Thus, we speculate that members of the HDGF family can anchor repair proteins to specific gene sets marked by histones to synergistically maintain genomic stability. Furthermore, we found that genes associated with the overlapping peaks of HDGF and H3K36me3 ChIP-seq are enriched in various oncogenic pathways, including the regulation of actin cytoskeleton, as well as signaling pathways for MAPK, insulin, AMPK, and VEGF, among others (Figure 7F). These pathways are related to various biological functions in CRC, such as proliferation, metastasis, apoptosis resistance, drug resistance, and angiogenesis. These results suggest that HDGF maintains the activity of these pathways by recognizing H3K36me3 and rapidly repairing the genes related to the above pathways. This process assists in maintaining the potential of CRC cells for proliferation, metastasis, anti-apoptosis, and angiogenesis, and promotes drug resistance.

In their latest study, Zhou et al. constructed a humanized anti-HDGF antibody with high affinity and specificity, demonstrating significant efficacy in treating EGFR-mutant patient-derived xenograft tumors when combined with tyrosine kinase inhibitor therapy [50]. These findings highlight the therapeutic potential of an anti-HDGF antibody in the treatment of cancer. Our results reveal that HDGF plays a critical role in facilitating HR-mediated DNA repair, thereby driving tumor progression and resistance to chemotherapy and PARP inhibitors in CRC. Therefore, it is reasonable to propose that an anti-HDGF antibody could also be effective in the treatment of CRC, particularly in combination with chemotherapy or PARP inhibitors. Importantly, conventional chemotherapy induces non-specific DNA damage and systemic toxicity, while PARP inhibitors exploit pre-existing HR defects but are ineffective in tumors with intact HR pathways. Compared with these treatment options, anti-HDGF therapy directly disrupts the upstream regulatory function of HDGF in DNA damage repair pathways. This targeted approach amplifies the efficacy of existing treatments (e.g., chemotherapy and PARP inhibitors) and minimizes systemic toxicity by reducing non-specific DNA damage.

## 5. Conclusions

The results of this study indicate that the expression of HDGF is crucial for maintaining normal function in CRC cells. When CRC cells encounter DNA damage, HDGF regulates the DDR and transmits damage signals by recognizing H3K36me3, thus recruiting CtIP to the DNA damage sites for HR-mediated repair to maintain genomic stability. Through this process, CRC cells sustain their potential for proliferation, metastasis, anti-apoptosis, angiogenesis, and drug resistance. This study provides novel insights into the mechanisms by which HDGF promotes the development of CRC. Based on this evidence, we propose a new strategy for targeting HDGF in combination with chemotherapy and PARP inhibitors in the treatment of CRC.

## Data Availability

All data generated are included in this article and its supplementary data files. Reagents and antibodies used in this study are detailed in the Supplementary Materials. The raw ChIP-Seq data supporting the conclusions of this article are available in the NCBI’s BioProject SRA database (PRJNA1122976).

## Supplementary Materials

The following supporting information can be downloaded at: https://www.mdpi.com/article/10.3390/biom15020282/s1, Figure S1: Schematic of HDGF gene editing sites and verification of DNA KO by Sanger sequencing, and exclusion of apoptosis and DNA damage potentially caused by the CRISPR/Cas9 system; Figure S2: Distribution of HDGF and H3K36me3 at TSS and Venn diagram of their overlapping peaks; Table S1: Reagents and antibodies; Table S2: Gene Ontology (GO) enrichment analysis of genes associated with HDGF and H3K36me3 overlapping peaks identified by ChIP-seq; Table S3: KEGG pathway enrichment analysis of genes associated with overlapping peaks of HDGF and H3K36me3 identified by ChIP-seq.

## References

[1] Dekker E., Tanis P.J., Vleugels J.L.A., Kasi P.M., Wallace M.B. (2019). Colorectal cancer. Lancet.

[2] Sung H., Ferlay J., Siegel R.L., Laversanne M., Soerjomataram I., Jemal A., Bray F. (2021). Global Cancer Statistics 2020: GLOBOCAN Estimates of Incidence and Mortality Worldwide for 36 Cancers in 185 Countries. CA Cancer J. Clin..

[3] Biller L.H., Schrag D. (2021). Diagnosis and Treatment of Metastatic Colorectal Cancer: A Review. JAMA.

[4] Jackson S.P., Bartek J. (2009). The DNA-damage response in human biology and disease. Nature.

[5] Lee Y.R., Kang G.S., Oh T., Jo H.J., Park H.J., Ahn J.O. (2023). DNA-Dependent Protein Kinase Catalytic Subunit (DNA-PKcs): Beyond the DNA Double-Strand Break Repair. Mol. Cells.

[6] Roos W.P., Thomas A.D., Kaina B. (2016). DNA damage and the balance between survival and death in cancer biology. Nat. Rev. Cancer.

[7] Nakamura H., Izumoto Y., Kambe H., Kuroda T., Mori T., Kawamura K., Yamamoto H., Kishimoto T. (1994). Molecular cloning of complementary DNA for a novel human hepatoma-derived growth factor. Its homology with high mobility group-1 protein. J. Biol. Chem..

[8] Enomoto H., Yoshida K., Kishima Y., Kinoshita T., Yamamoto M., Everett A.D., Miyajima A., Nakamura H. (2002). Hepatoma-derived growth factor is highly expressed in developing liver and promotes fetal hepatocyte proliferation. Hepatology.

[9] Lepourcelet M., Tou L., Cai L., Sawada J.I., Lazar A.J., Glickman J.N., Williamson J.A., Everett A.D., Redston M., Fox E.A. (2005). Insights into developmental mechanisms and cancers in the mammalian intestine derived from serial analysis of gene expression and study of the hepatoma-derived growth factor (HDGF). Development.

[10] LeBlanc M.E., Wang W., Chen X., Ji Y., Shakya A., Shen C., Zhang C., Gonzalez V., Brewer M., Ma J. (2016). The regulatory role of hepatoma-derived growth factor as an angiogenic factor in the eye. Mol. Vis..

[11] Oliver J.A., Al-Awqati Q. (1998). An endothelial growth factor involved in rat renal development. J. Clin. Investig..

[12] Zhuang Z., Mei G., Liu W., Chen Y., Zeng J., Zhang W., Yao G., Wang X. (2015). Hepatoma-derived growth factor-2 is highly expressed during development and in spinal cord injury. Mol. Med. Rep..

[13] Enomoto H., Nakamura H., Liu W., Yoshida K., Okuda Y., Imanishi H., Saito M., Shimomura S., Hada T., Nishiguchi S. (2009). Hepatoma-derived growth factor is induced in liver regeneration. Hepatol. Res..

[14] Zuo X., Chen Z., Gao W., Zhang Y., Wang J., Wang J., Cao M., Cai J., Wu J., Wang X. (2020). M6A-mediated upregulation of LINC00958 increases lipogenesis and acts as a nanotherapeutic target in hepatocellular carcinoma. J. Hematol. Oncol..

[15] Wang Q., Chen C., Ding Q., Zhao Y., Wang Z., Chen J., Jiang Z., Zhang Y., Xu G., Zhang J. (2020). METTL3-mediated m(6)A modification of HDGF mRNA promotes gastric cancer progression and has prognostic significance. Gut.

[16] Hsu S.S., Chen C.H., Liu G.S., Tai M.H., Wang J.S., Wu J.C., Kung M.L., Chan E.C., Liu L.F. (2012). Tumorigenesis and prognostic role of hepatoma-derived growth factor in human gliomas. J. Neurooncol..

[17] Yang Y., Ma Y., Gao H., Peng T., Shi H., Tang Y., Li H., Chen L., Hu k., Han A. (2021). A novel HDGF-ALCAM axis promotes the metastasis of Ewing sarcoma via regulating the GTPases signaling pathway. Oncogene.

[18] Chen W., Zhou Y., Wu G., Sun P. (2021). CCNI2 promotes the progression of human gastric cancer through HDGF. Cancer Cell Int..

[19] Zhao J., Ma M.Z., Ren H., Liu Z., Edelman M.J., Pan H., Mao L. (2013). Anti-HDGF targets cancer and cancer stromal stem cells resistant to chemotherapy. Clin. Cancer Res..

[20] Daugaard M., Baude A., Fugger K., Povlsen L.K., Beck H., Sørensen C.S., Petersen N.H., Sorensen P.H., Lukas C., Bartek J. (2012). LEDGF (p75) promotes DNA-end resection and homologous recombination. Nat. Struct. Mol. Biol..

[21] Baude A., Aaes T.L., Zhai B., AI-Nakouzi N., Oo H.Z., Daugaard M., Rohde M., Jäättelä M. (2016). Hepatoma-derived growth factor-related protein 2 promotes DNA repair by homologous recombination. Nucleic Acids Res..

[22] Zhang Z., Samsa W.E., De Y., Zhang F., Reizes O., Almasan A., Gong Z. (2023). HDGFRP3 interaction with 53BP1 promotes DNA double-strand break repair. Nucleic Acids Res..

[23] Wang H., Farnung L., Dienemann C., Cramer P. (2020). Structure of H3K36-methylated nucleosome-PWWP complex reveals multivalent cross-gyre binding. Nat. Struct. Mol. Biol..

[24] Yu G., Wang L.G., He Q.Y. (2015). ChIPseeker: An R/Bioconductor package for ChIP peak annotation, comparison and visualization. Bioinformatics.

[25] Yu G., Wang L.G., Han Y., He Q.Y. (2012). clusterProfiler: An R package for comparing biological themes among gene clusters. Omics.

[26] Rogakou E.P., Pilch D.R., Orr A.H., Ivanova V.S., Bonner W.M. (1998). DNA double-stranded breaks induce histone H2AX phosphorylation on serine 139. J. Biol. Chem..

[27] Blackford A.N., Jackson S.P. (2017). ATM, ATR, and DNA-PK: The Trinity at the Heart of the DNA Damage Response. Mol. Cell.

[28] Wu W.Y., Wang Z.X., Li T.S., Ding X.Q., Liu Z.H., Yang J., Fang L., Kong L.D. (2022). SSBP1 drives high fructose-induced glomerular podocyte ferroptosis via activating DNA-PK/p53 pathway. Redox Biol..

[29] Wu L., He Y., Hu Y., Lu H., Cao Z., Yi X., Wang J. (2019). Real-time surface plasmon resonance monitoring of site-specific phosphorylation of p53 protein and its interaction with MDM2 protein. Analyst.

[30] Ma D., Chen X., Zhang P.Y., Zhang H., Wei L.J., Hu S., Tang J.Z., Zhou M.T., Xie C., Ou R. (2018). Upregulation of the ALDOA/DNA-PK/p53 pathway by dietary restriction suppresses tumor growth. Oncogene.

[31] Zhang X.P., Liu F., Cheng Z., Wang W. (2009). Cell fate decision mediated by p53 pulses. Proc. Natl. Acad. Sci. USA.

[32] Scully R., Panday A., Elango R., Willis N.A. (2019). DNA double-strand break repair-pathway choice in somatic mammalian cells. Nat. Rev. Mol. Cell Biol..

[33] Rivera-Calzada A., Spagnolo L., Pearl L.H., LIorca O. (2007). Structural model of full-length human Ku70-Ku80 heterodimer and its recognition of DNA and DNA-PKcs. EMBO Rep..

[34] Shi W., Feng Z., Zhang J., Gonzalez-Suarez I., Vanderwaal R.P., Wu X., Powell S.N., Roti Roti J.L., Gonzalo S., Zhang J. (2010). The role of RPA2 phosphorylation in homologous recombination in response to replication arrest. Carcinogenesis.

[35] Symington L.S. (2016). Mechanism and regulation of DNA end resection in eukaryotes. Crit. Rev. Biochem. Mol. Biol..

[36] Sun Z., Zhang Y., Jia J., Fang Y., Tang Y., Wu H., Fang D. (2020). H3K36me3, message from chromatin to DNA damage repair. Cell Biosci..

[37] Aymard F., Bugler B., Schmidt C.K., Guillou E., Caron P., Briois S., Iacovoni J.S., Daburon V., Miller K.M., Jackson S.P. (2014). Transcriptionally active chromatin recruits homologous recombination at DNA double-strand breaks. Nat. Struct. Mol. Biol..

[38] Lahav G., Rosenfeld N., Sigal A., Geva-Zatorsky N., Levine A.J., Elowitz M.B., Alon U. (2004). Dynamics of the p53-Mdm2 feedback loop in individual cells. Nat. Genet..

[39] Sasaki Y., Negishi H., Idogawa M., Yokota I., Koyama R., Kusano M., Suzuki H., Fujita M., Maruyama R., Toyota M. (2011). p53 negatively regulates the hepatoma growth factor HDGF. Cancer Res..

[40] Bremer S., Klein K., Sedlmaier A., Abouzied M., Gieselmann V., Franken S. (2013). Hepatoma-derived growth factor and nucleolin exist in the same ribonucleoprotein complex. BMC Biochem..

[41] Zhao J., Yu H., Lin L., Tu J., Cai L., Chen Y., Zhong F., Lin C., He F., Yang F. (2011). Interactome study suggests multiple cellular functions of hepatoma-derived growth factor (HDGF). J. Proteomics.

[42] Murphy A.K., Fitzgerald M., Ro T., Kim J.H., Rabinowitsch A.I., Chowdhury D., Schildkraut C.L., Borowiec J.A. (2014). Phosphorylated RPA recruits PALB2 to stalled DNA replication forks to facilitate fork recovery. J. Cell Biol..

[43] Liedtke V., Schröder C., Roggenbuck D., Weiss R., Stohwasser R., Schierack P., Rödiger S., Schenk L. (2021). LEDGF/p75 Is Required for an Efficient DNA Damage Response. Int. J. Mol. Sci..

[44] Chou J., Kaller M., Jaeckel S., Rokavec M., Hermeking H. (2022). AP4 suppresses DNA damage, chromosomal instability and senescence via inducing MDC1/Mediator of DNA damage Checkpoint 1 and repressing MIR22HG/miR-22-3p. Mol. Cancer.

[45] Wu C., Shi W., Zhang S. (2023). ZEB1 promotes DNA homologous recombination repair and contributes to the 5-Fluorouracil resistance in colorectal cancer. Am. J. Cancer Res..

[46] Hsieh C.C., Hsu S.H., Lin C.Y., Liaw H.J., Li T.W., Jiang K.Y., Chiang N.J., Chen S.H., Lin B.W., Chen P.C. (2022). CHK2 activation contributes to the development of oxaliplatin resistance in colorectal cancer. Br. J. Cancer.

[47] Arena S., Corti G., Durinikova E., Montone M., Reilly N.M., Russo M., Lorenzato A., Arcella P., Lazzari L., Rospo G. (2020). A Subset of Colorectal Cancers with Cross-Sensitivity to Olaparib and Oxaliplatin. Clin. Cancer Res..

[48] Li J., Ahn J.H., Wang G.G. (2019). Understanding histone H3 lysine 36 methylation and its deregulation in disease. Cell Mol. Life Sci..

[49] Hu J., Wang Y. (2013). p53 and the PWWP domain containing effector proteins in chromatin damage repair. Cell Dev. Biol..

[50] Zhou C.Q., Li A., Ri K., Sultan A.S., Ren H. (2024). Anti-HDGF Antibody Targets EGFR Tyrosine Kinase Inhibitor-Tolerant Cells in NSCLC Patient-Derived Xenografts. Cancer Res. Commun..

