# Supplementary material for: HDGF Knockout Suppresses Colorectal Cancer Progression and Drug Resistance by Modulating the DNA Damage Response
Source: Biomolecules. 2025 Feb 14;15(2):282. doi: 10.3390/biom15020282 (PMC11853149; doi:10.3390/biom15020282)
Supplement: Supplementary file 1 [file biomolecules-15-00282-s001.zip › Supplementary figures.pdf]

Supplementary figures

HDGF Knockout Suppresses Colorectal Cancer Progression and Drug Resistance by Modulating the DNA Damage Response

Authors

Riya Su 1,†, Qin Wang 1,†, Qun Hu 2, Wendurige 3, Kexin Li 3, Changshan Wang 3,* and Liang Tao 1,*

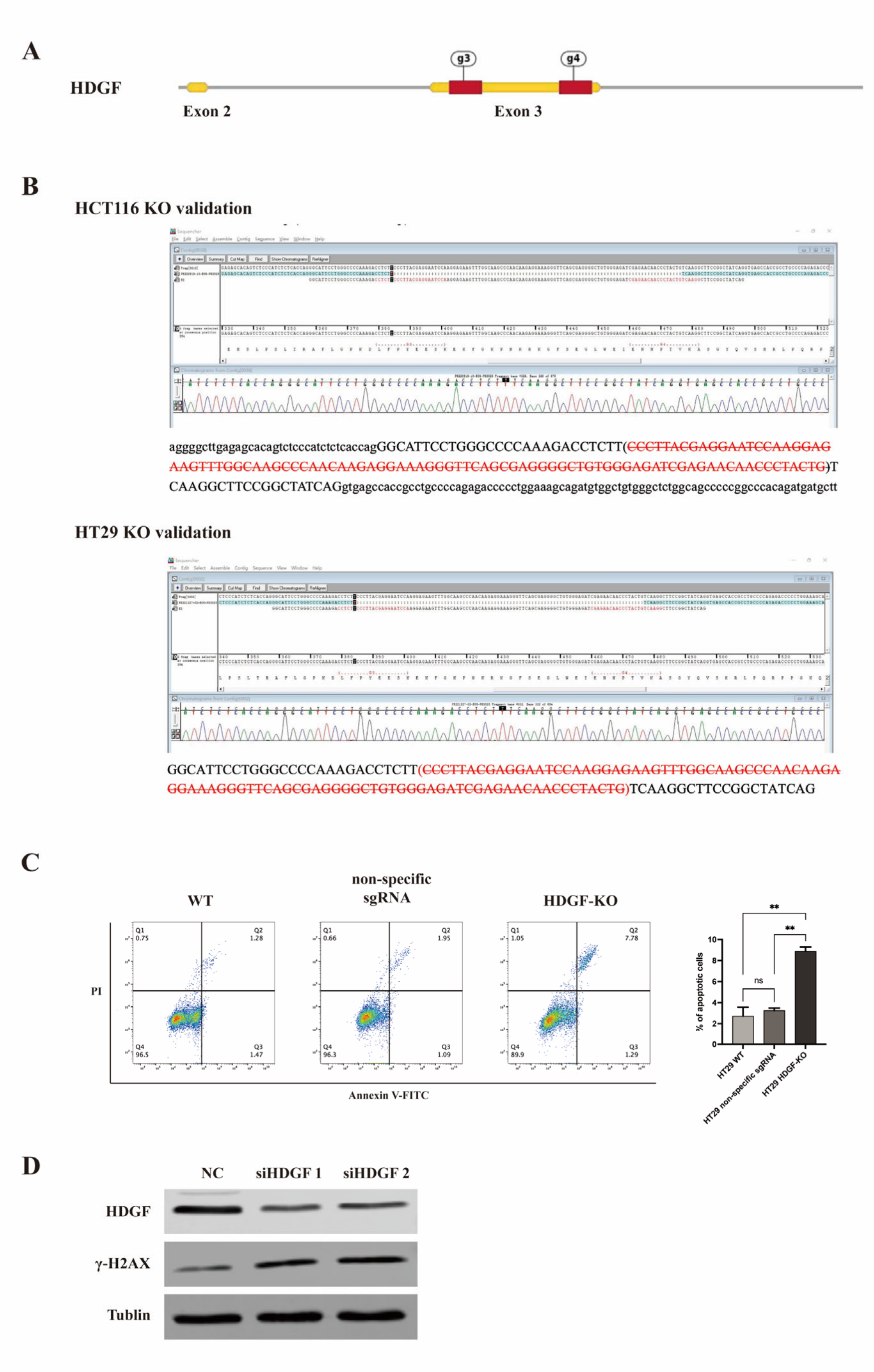

Figure S1: Schematic of HDGF gene editing sites and verification of DNA KO by Sanger sequencing, and exclusion of apoptosis and DNA damage potentially caused by the CRISPR/Cas9 system. (A) Schematic representation of HDGF gene editing sites (E3 represents exon 3; g3 and g4 represent two gRNAs). (B) Sanger sequencing was used to verify HDGF KO in HCT116 (top) and HT29 cells (bottom). (C) Comparing apoptosis rates among HT29 WT cells, HT29 cells transfected with non-specific sgRNA, and HT29 HDGF-KO cells. (D) IB detection of γ-H2AX expression in HT29 WT cells following transfection with siRNAs (n=3).

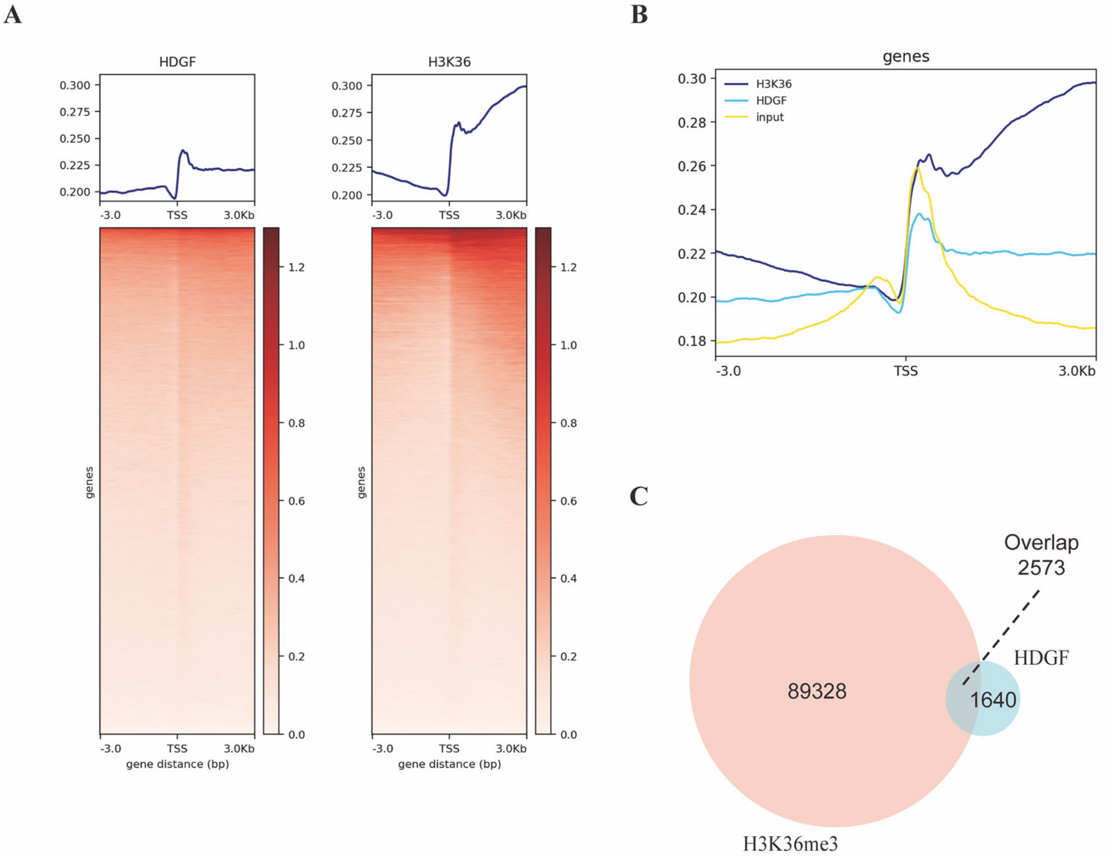

Fig S2. Distribution of HDGF and H3K36me3 at TSS and Venn diagram of their overlapping peaks. (A) Heatmaps illustrating the distribution of HDGF and H3K36me3 on the TSS in HCT116 WT cells. **(B)** Peak graph showing the distribution of HDGF and H3K36me3 on the TSS and their adjacent 3 kb regions upstream and downstream in HCT116 WT cells. **(C)** Venn diagram illustrating the overlapping peaks between HDGF and H3K36me3 ChIP-seq in HCT116 WT cells.
