# Supplementary material for: HDGF Knockout Suppresses Colorectal Cancer Progression and Drug Resistance by Modulating the DNA Damage Response
Source: Biomolecules. 2025 Feb 14;15(2):282. doi: 10.3390/biom15020282 (PMC11853149; doi:10.3390/biom15020282)
Supplement: Supplementary file 1 [file biomolecules-15-00282-s001.zip › Table S1.dox.pdf]

Supplementary Table S1: Reagents and antibodies

**Reagents**	**Catalog number**	**Manufacturer**	
Camptothecin	HY-16560	Med Chem Express, USA	
5-Fluorouracil	S1209	Selleck, Houston, TX, USA	
Oxaliplatin	S1224	Selleck, Houston, TX, USA	
Niraparib	S2741	Selleck, Houston, TX, USA	
NU7441	HY-11006	Med Chem Express, USA	
Ku55933	S1092	Selleck, Houston, TX, USA	
**Antibodies**	**Catalog number**	**Manufacturer**	**Dilution ratio**
HDGF	42105	Cell Signaling Technology, Danvers, MA, USA	IB 1:1000
Tubulin	10094	Proteintech, USA	IB 1:2000
Anti-rabbit IgG (H+L)	5151P	Cell Signaling Technology, Danvers, MA, USA	IB 1:15000
γ-H2AX	ab303656	Abcam, Cambridge, UK	IF 1:500
DNA-PK phospho S2056	ab124918	Abcam, Cambridge, UK	IB 1:5000
DNA-PK	ab32566	Abcam, Cambridge, UK	IB 1:1000
ATM phospho S1981	ab81292	Abcam, Cambridge, UK	IB 1:50000
ATM	ab32420	Abcam, Cambridge, UK	IB 1:1000
ATR phospho T1989	ab223258	Abcam, Cambridge, UK	IB 1:1000
ATR	13934	Cell Signaling Technology, Danvers, MA, USA	IB 1:1000
CHK1 phospho Ser345	2348	Cell Signaling Technology, Danvers, MA, USA	IB 1:1000
CHK1	25887	Proteintech, USA	IB 1:1000
CHK2 phospho Thr68	2197	Cell Signaling Technology, Danvers, MA, USA	IB 1:1000
CHK2	13954	Proteintech, USA	IB 1:1000
Cleaved-PARP	PTM-5272	PTM BIO, Hangzhou, China	IB 1:1000
p53 phospho S15	ab223868	Abcam, Cambridge, UK	IB 1:5000
p53 phospho S37	ab182164	Abcam, Cambridge, UK	IB 1:5000
p53	10442	Proteintech, USA	IB 1:5000
GAPDH	10494	Proteintech, USA	IB 1:5000
HDGF	11344	Proteintech, USA	IP 4 μg/per IP
Ku70	ab92450	Abcam, Cambridge, UK	IB 1:1000
Ku80	ab80592	Abcam, Cambridge, UK	IB 1:1000
RPA2	ab76420	Abcam, Cambridge, UK	IB 1:1000, IF 1:50

CtIP	MA1-23304	Invitrogen, Carlsbad, CA, USA	IF 1:100
CtIP	sc-271339	Santa Cruz, Dallas, TX, USA	IP 8 µg per IP
CtIP	9201	Cell Signaling Technology, Danvers, MA, USA	IB 1:1000
MRE11	4847	Cell Signaling Technology, Danvers, MA, USA	IB 1:1000
RAD50	29390	Proteintech, USA	IB 1:2000
NBS1	14956	Cell Signaling Technology, Danvers, MA, USA	IB 1:1000
Rabbit IgG control	30000	Proteintech, USA	IP 4 µg per IP
Mouse IgG control	5415	Cell Signaling Technology, Danvers, MA, USA	IP 8 µg per IP
goat anti-rabbit IgG Alexa Fluor-488	4412	Cell Signaling Technology, Danvers, MA, USA	IF 1:500
goat anti-mouse IgG Alexa Fluor-647	4410	Cell Signaling Technology, Danvers, MA, USA	IF 1:500
goat anti-mouse IgG Alexa Fluor-488	4408	Cell Signaling Technology, Danvers, MA, USA	IF 1:500
