# Supplementary figures and images for: HDGF Knockout Suppresses Colorectal Cancer Progression and Drug Resistance by Modulating the DNA Damage Response
Source: Biomolecules. 2025 Feb 14;15(2):282. doi: 10.3390/biom15020282 (PMC11853149; doi:10.3390/biom15020282)

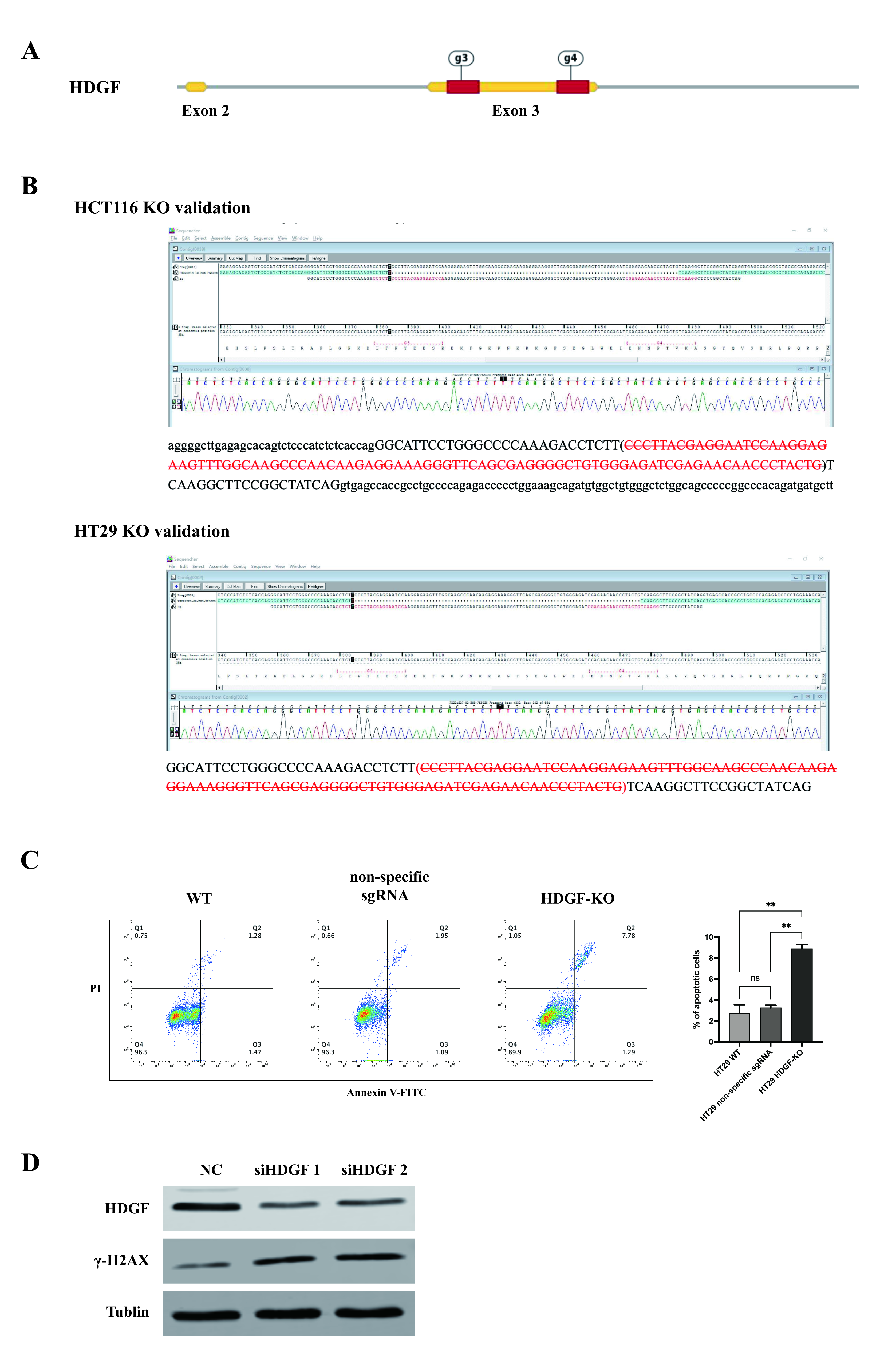

Supplement: Supplementary file 1 [file biomolecules-15-00282-s001.zip › Fig S1.tif]

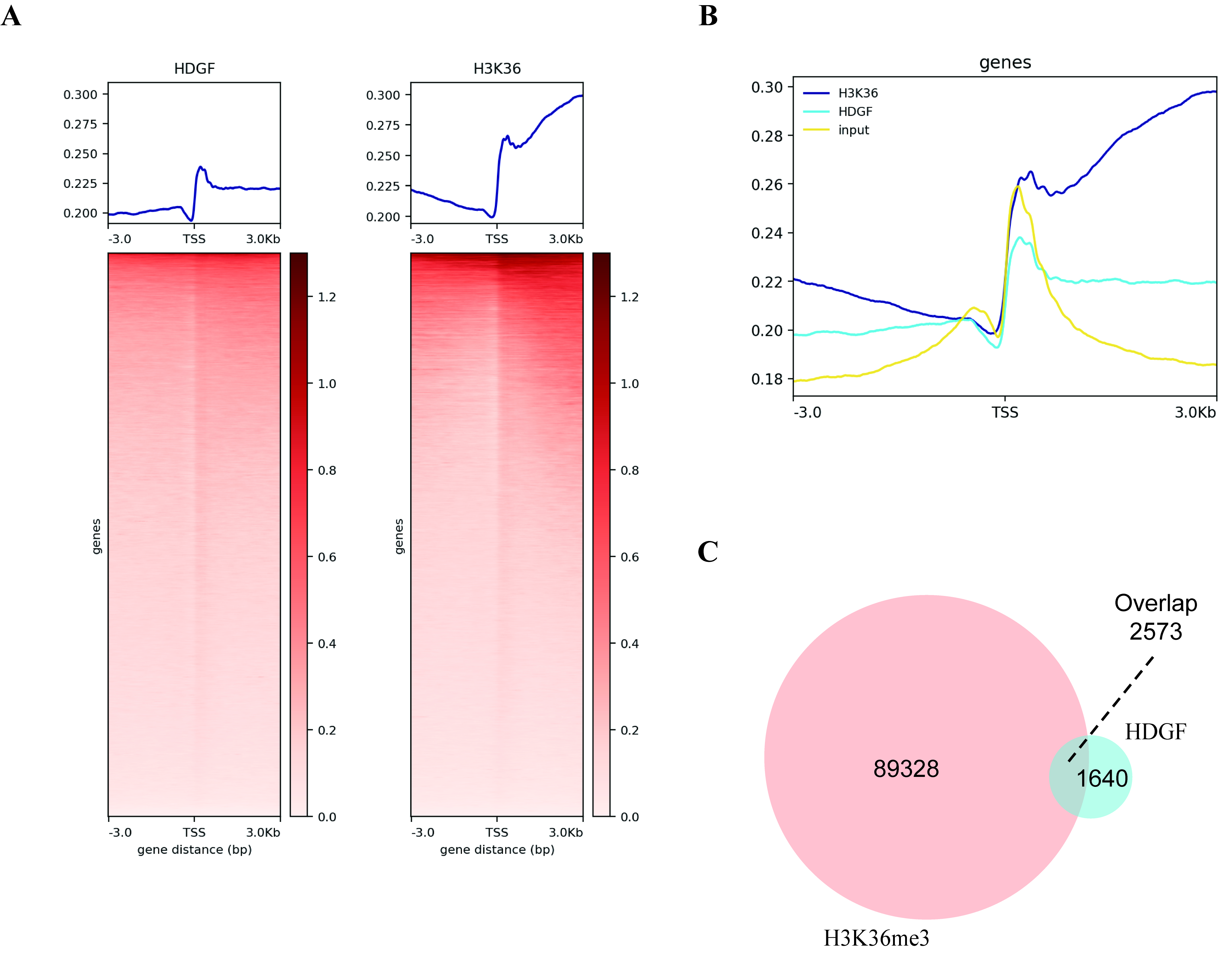

Supplement: Supplementary file 1 [file biomolecules-15-00282-s001.zip › Fig S2.tif]

Fig. 1D

HCT116 WT vs HCT116 KO

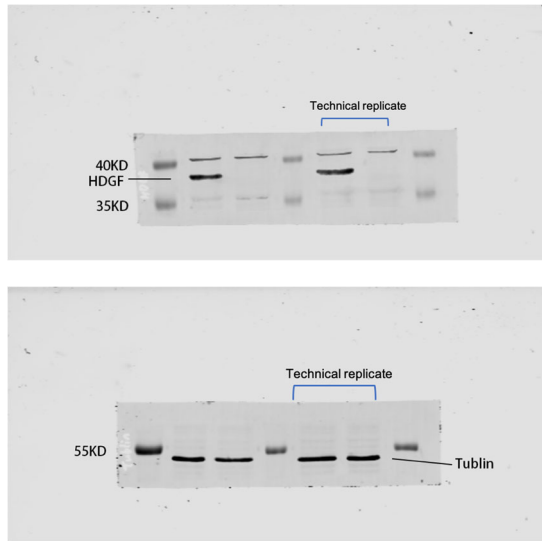

HT29 WT vs HT29 KO

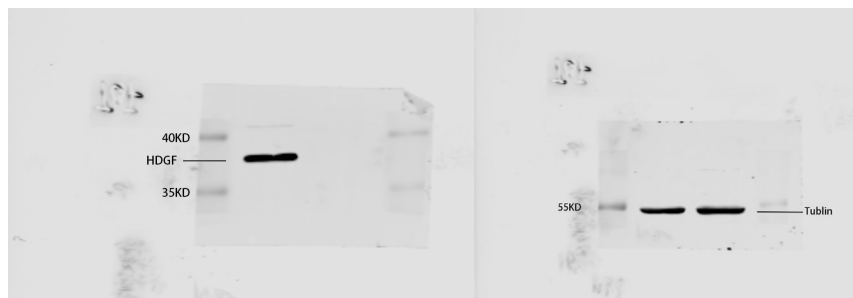

Fig. 2B

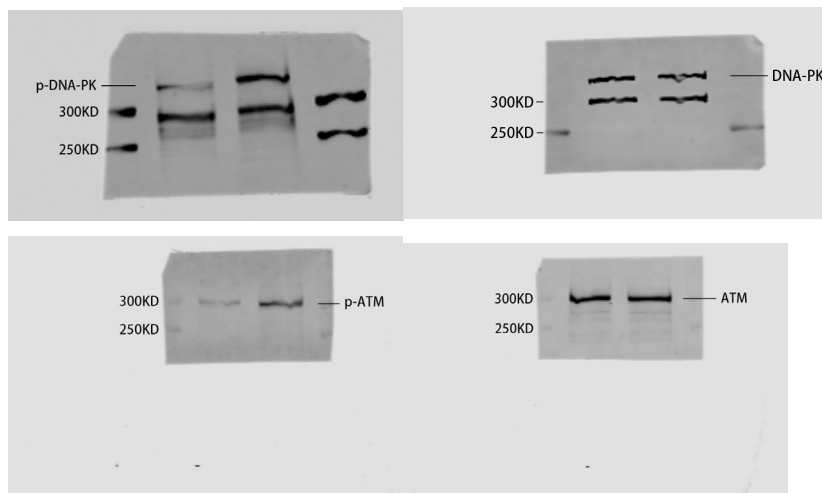

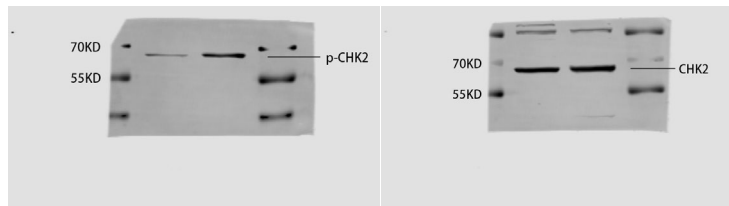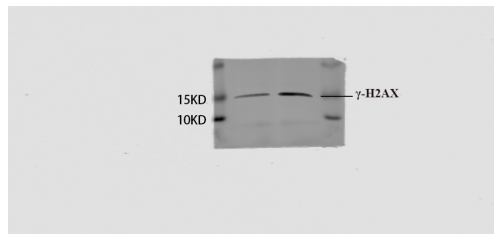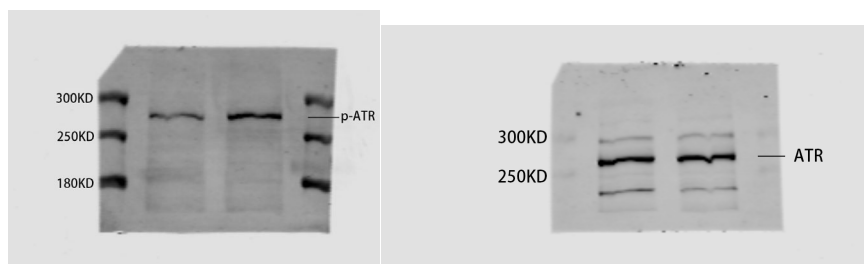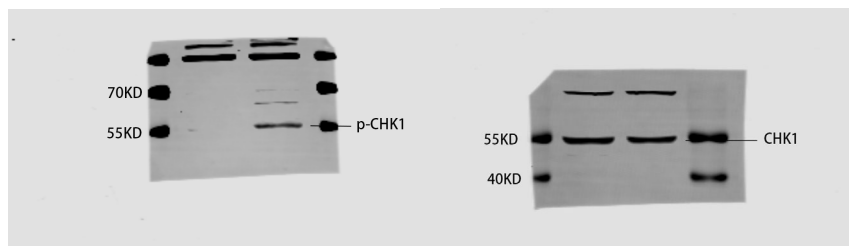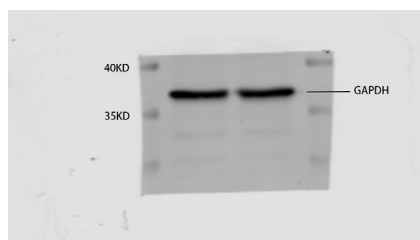

Fig. 2C

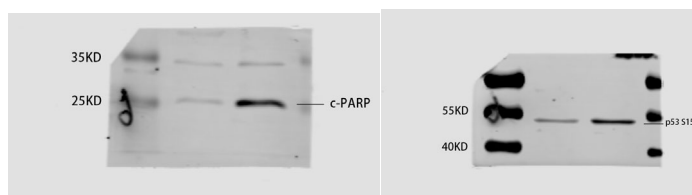

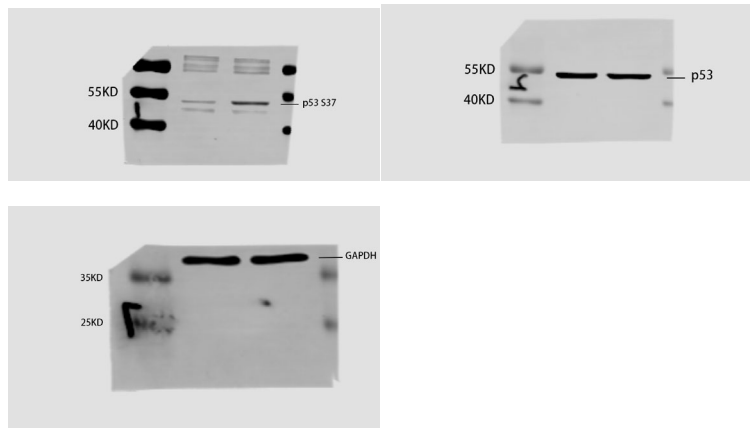

Fig. 2D

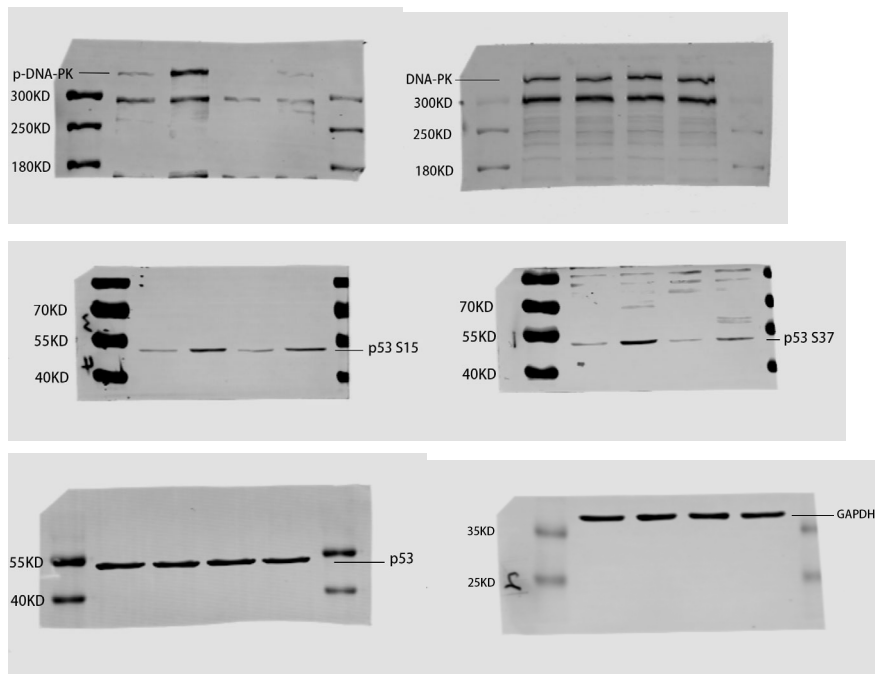

Fig. 2E

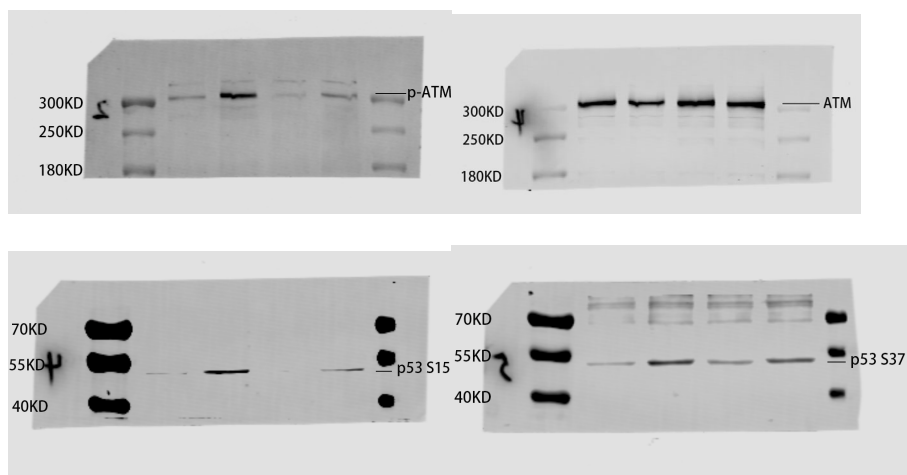

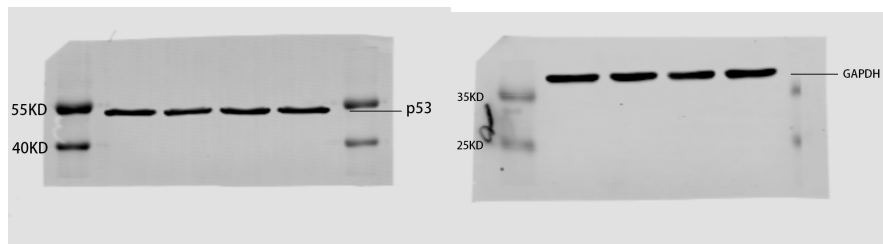

Fig. 3A

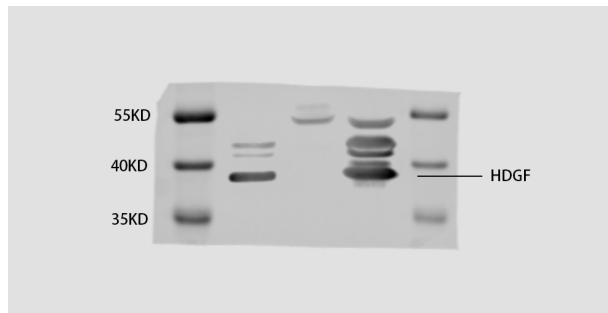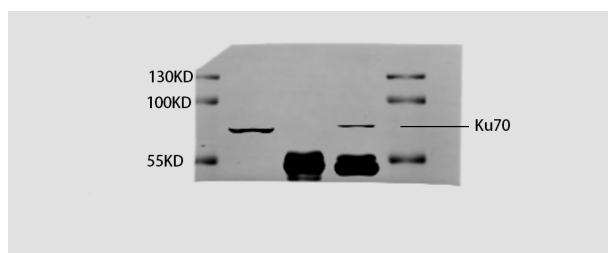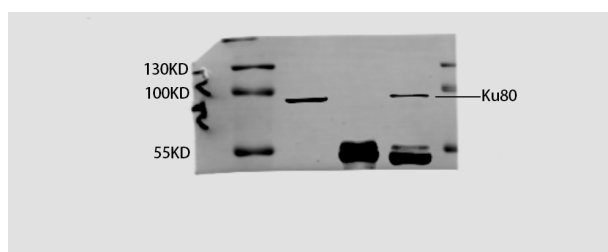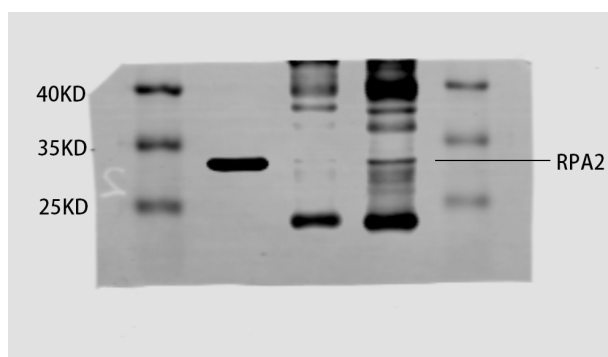

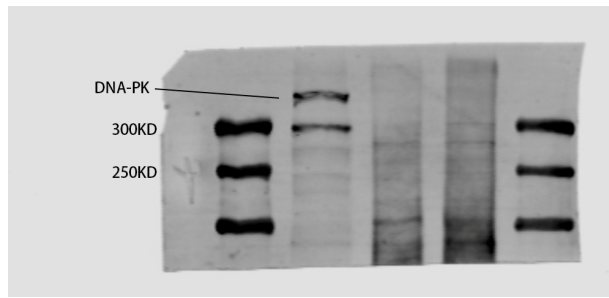

Fig. 4A

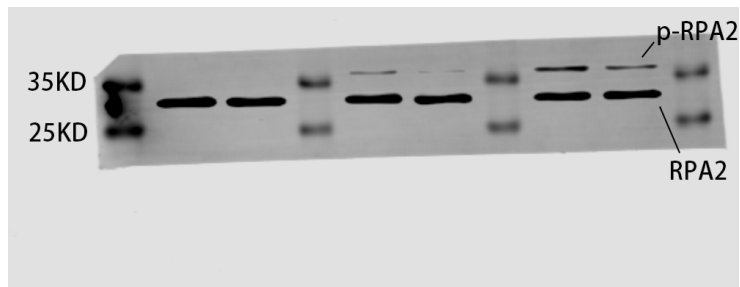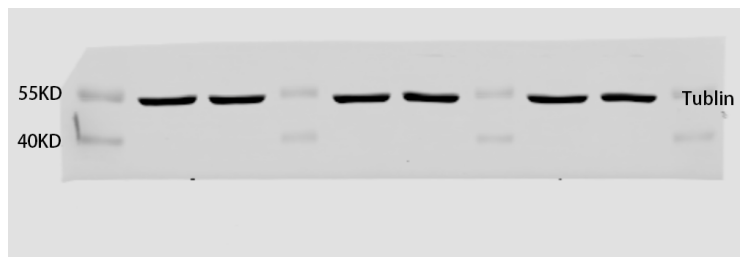

Fig. 4B

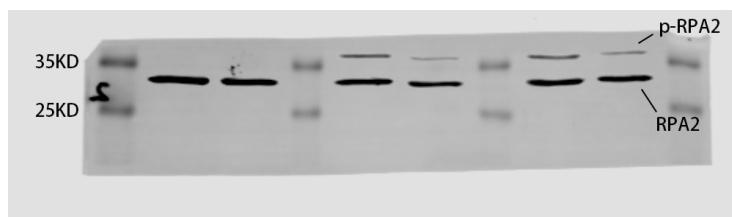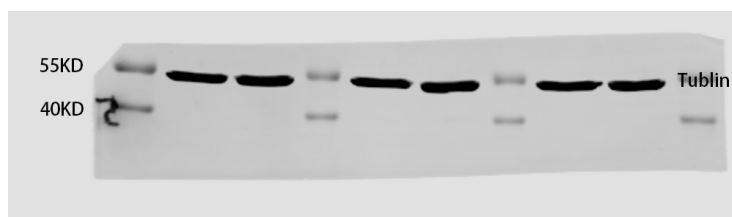

Fig. 4E  
HCT116

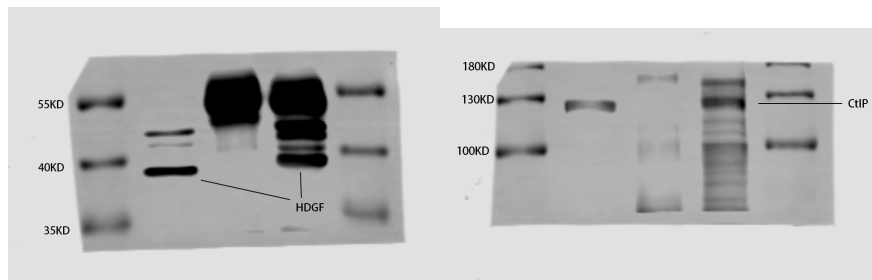

HT29

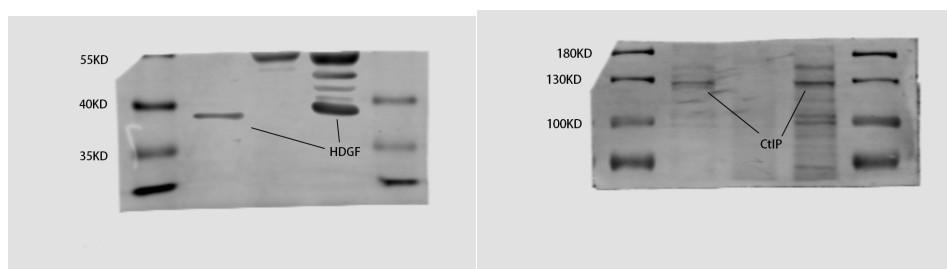

Fig. 7A  
HCT116

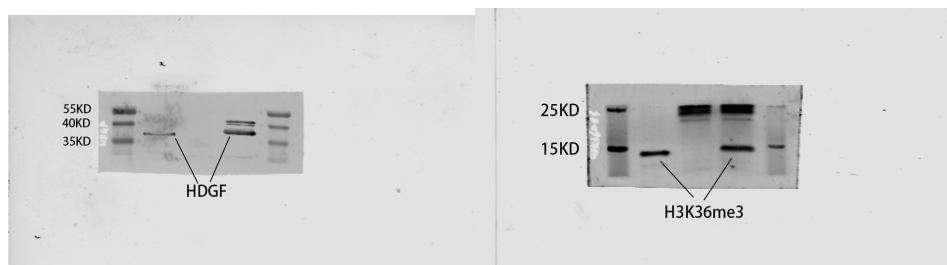

HT29

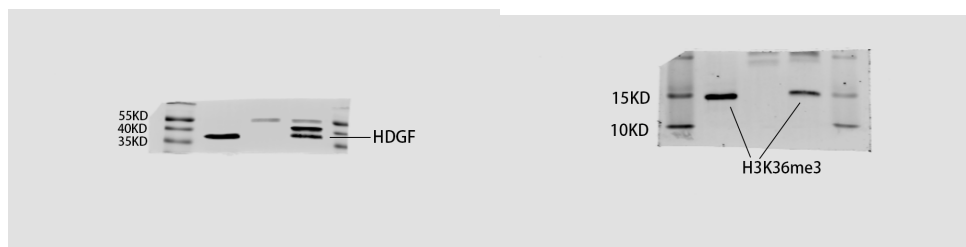

Supplement: Supplementary file 1 [file biomolecules-15-00282-s001.zip › WB_raw data.pdf]
